# Distinguished biomimetic dECM system facilitates early detection of metastatic breast cancer cells

**DOI:** 10.1002/btm2.10597

**Published:** 2023-09-01

**Authors:** Bowen Weng, Mei Li, Weilai Zhu, Jing Peng, Xufeng Mao, Yanan Zheng, Chi Zhang, Senhao Pan, Haijiao Mao, Jiyuan Zhao

**Affiliations:** ^1^ Zhejiang Key Laboratory of Pathophysiology School of Medicine, Ningbo University Ningbo Zhejiang China; ^2^ Key Laboratory of Precision Medicine for Atherosclerotic Diseases of Zhejiang Province The First Affiliated Hospital of Ningbo University Ningbo Zhejiang China; ^3^ Department of Orthopaedic Surgery The First Affiliated Hospital of Ningbo University Ningbo Zhejiang China

**Keywords:** breast cancer bone metastasis, decellularized extracellular matrix (ECM), early metastatic diagnosis, in vitro tumor model, native niches

## Abstract

Breast cancer is the most prevalent malignant tumor affecting women's health. Bone is the most common distant metastatic organ, worsening the quality of life and increasing the mortality of patients. Early detection of breast cancer bone metastasis is urgent for halting disease progression and improving tumor prognosis. Recently, extracellular matrix (ECM) with biomimetic tissue niches opened a new avenue for tumor models in vitro. Here, we developed a biomimetic decellularized ECM (dECM) system to recapitulate bone niches at different situations, bone mimetic dECM from osteoblasts (BM‐ECM) and bone tumor mimetic dECM from osteosarcoma cells (OS‐ECM). The two kinds of dECMs exhibited distinct morphology, protein composition, and distribution. Interestingly, highly metastatic breast cancer cells tended to adhere and migrate on BM‐ECM, while lowly metastatic breast cancer cells preferred the OS‐ECM niche. Epithelial‐to‐mesenchymal transition was a potential mechanism to initiate the breast cancer cell migration on different biomimetic dECMs. Importantly, in the nude mice model, the dECM system captured metastatic breast cancer cells as early as 10 days after orthotopic transplantation in mammary gland pads, with higher signal on BM‐ECM than that on OS‐ECM. Collectively, the biomimetic dECM system might be a promising tumor model to distinguish the metastatic ability of breast cancer cells in vitro and to facilitate early detection of metastatic breast cancer cells in vivo, contributing to the diagnosis of breast cancer bone metastasis.

**Translational Impact Statements**

Bone is the most frequent organ of breast cancer metastasis. Early diagnosis is crucial for clinical treatment and prognostic survival. Here, we developed a biomimetic decellularized extracellular matrix (dECM) system with bone mimetic niche and bone tumor mimetic niche. The constructed biomimetic dECM system is conductive to identify the metastatic ability of breast cancer cells and capture the early metastatic breast cancer cells in vivo. The dECM system will promote the early diagnosis of breast cancer bone metastasis, holding great potential for clinical translation.

## 1 INTRODUCTION

Breast cancer was the most common malignant cancer affecting women,^1^, ^2^ while bone metastasis was the most frequent complication of it.^3^ More than 50% of breast cancer patients showed bone metastasis with serious bone events, such as pain, pathological fractures, nerve compression syndromes, and hypercalcemia.^4^ Most importantly, once the bone metastasis occurred, they were virtually incurable and resulted in high rate of mortality.^4^, ^5^ Therefore, early detection of metastasis before tumor cell colonization in bone should be essential for the prognosis and the survival of breast cancer patients. Recently, many efforts have been made for the early diagnosis of breast cancer bone metastasis, especially the detection of circulating tumor cells (CTCs).^6^ Even though the disadvantages of CTCs still limit its application in clinic, including low numbers in blood,^7^ low sensitivity, and specificity of CTCs biomarkers. Moreover, CTCs might exist in blood for a long time without colonization in bone tissue.^3^, ^8^

The interactions between malignant cells and surrounding microenvironment played an essential role during cancer progression and metastasis. For distant organ metastasis of cancer, Stephen Paget first proposed the “seed‐and‐soil” hypothesis as early as 1889, and increasing evidences demonstrated the importance of the target organ microenvironment with the selective advantage of cells to grow.^9^ The extracellular matrix (ECM) was the major component of tissue microenvironment with crucial ECM proteins and three‐dimensional (3D) network structure. It provided biochemical and biophysical signals as the “soil” of the organ microenvironment, deciding specific tumor cell activities.^10^ Some researchers reported that ECM from different tissues provided distinguished microenvironment for breast cancer metastasis.^11^ Moreover, the alteration of ECM components and structure with distinguished tissue niches was demonstrated to affect the cancer metastasis. For example, ECM protein HAPLN1 was significantly deficient in aging skin, resulting in the alteration of the matrix's arrangement structure and reduction of the skin's flexibility, compared to the ECM from young dermal fibroblasts.^12^ The altered ECM in aging skin significantly promotes the migration of melanoma cells with optical tissue microenvironment.^12^ Cooper et al.^13^ reported that paclitaxel chemotherapy increased the secretion of LOX from CD8 T cells, which remodeled the ECM in the lung and promoted breast cancer metastasis. Conventional metastasis surrogates (Transwell migration assays, wound healing) lacked the complex ECM components and structure, which were hard to clarify the real progression of cancer metastasis in vivo. Development of biomimetic ECM scaffolds as in vitro tumor models facilitated cancer metastasis studies with the interaction of cancer cells and matrix microenvironment.

In the past decades, decellularized ECM (dECM) from tissues or cells as bioactive materials have been extensively applied for tissue engineering and regenerative medicine. For bone tissue engineering, such biomaterials were demonstrated to mimic the complex components and structure of native tissue in vitro and promoted bone regeneration.^14^ Our previous studies have generated such dECM scaffolds from both tissue (small intestinal submucosa) and cells (osteoblasts or fibroblasts). All these biomaterials with bone mimetic microenvironment showed positive effects on bone regeneration and exhibited respective unique matrisome.^15^, ^16^, ^17^ Compared with tissue‐derived ECM, cell‐derived ECM showed several advantages, including faster, easier operation, economical, and more preservation of ECM composition because of less vigorous decellularization.

While dECM‐based biomaterials have been extensively applied for tissue engineering and regenerative medicine, only recently have these complex biomaterials been translated to in vitro tumor modeling.^18^, ^19^, ^20^, ^21^ The advantages of dECM have attracted much attention in cancer research, including structural support, suitable microenvironment, and bioactive factors.^22^ Decellularized ECM from both healthy peritumoral tissue and cancer tissue were reported to study the mechanisms of cancer progression because of their mimetic native tissues.^23^, ^24^, ^25^ Decellularized normal porcine livers and lungs were used to generate biomatrix scaffolds as metastatic soil and were proved to be beneficial of cancer cell invasion, colonization, and proliferation with functional readouts regarding epithelial‐to‐mesenchymal transition (EMT) and chemoresistance.^24^ Piccoli et al. reported that the pathologic dECM from colorectal cancer decreased angiogenic potential compared with healthy tissue.^25^ Moreover, Lv et al. developed 3D dECM scaffolds with different stiffness to mimic the microenvironment of breast cancer, including matrix stiffness, components, and structure of ECM. Lysyl oxidase (LOX) expression levels in dECM were essential for the diverse stiffness of breast cancer matrix and drug resistance.^26^ Therefore, dECM from bone‐associated cells might serve as a potent “soil” to study breast cancer bone metastasis.

The aim of the study was to develop a biomimetic dECM system to distinguish the metastatic ability of breast cancer cells and capture the highly metastatic breast cancer cells. Our previous studies have proved that dECM from osteoblasts with dense structure and high components of core matrisome proteins provided excellent bone mimetic microenvironment,^27^, ^28^ which was also introduced here to mimic normal bone matrix. Meanwhile, dECM derived from osteosarcoma cells was introduced to mimic a bone tumor matrix. The two kinds of dECM representing different bone microenvironments were applied as a system to distinguish the metastatic activities of breast cancer cells. Cell activities of high‐metastatic breast cancer cells or low‐metastatic breast cancer cells on different dECM biomaterials were examined in vitro. Besides, early detection of metastatic breast cancer cells with the distinguished biomimetic ECM system was assessed in vivo.

## 2 MATERIALS AND METHODS

### 2.1 Cell culture and generation of decellularized ECM

MC3T3‐E1 subclone 14, MG63, MCF7, and HCC1937 cells were provided by Cell Bank, Chinese Academy of Sciences. MDA‐MB‐231 cells were purchased from BLUEFBIO™ (BFN608008564). The basic media supplemented with 10% fetal bovine serum (FBS) and penicillin (100 U/mL)‐streptomycin (0.1 mg/mL) were prepared for cell culture as follows: α‐MEM (MC3T3‐E1, MG63, and MCF7), RPMI1640 (HCC1937 and 4T1), DMEM (without sodium pyruvate, 10‐040‐CVRC, Corning) (MDA‐MB‐231). MC3T3‐E1 subclone 14 cells were isolated from newborn mouse calvaria. MG‐63 osteosarcoma cells were from a male. MC3T3‐E1 and MG63 were used to generate bone mimetic or bone tumor mimetic dECM microenvironment. We do not know yet how using male source‐derived ECM may be different from female sources. The breast cancer cells 4T1 were from female mice, while MDA‐MB‐231, MCF7, and HCC1937 were from female human. The purpose of the distinguished biomimetic ECM system was to detect the potential risk of bone metastasis in breast cancer mainly occurred in women.

The MC3T3‐E1 and MG63 cells were seeded on tissue culture plates at a density of 1 × 10^4^/cm^2^. The cells were cultured for 5 days in complete media and for another 7 days with ascorbic acid (50 μg/mL). During this period, the cells were changed with fresh media every other day. The decellularization process was performed as previously reported.^29^ Briefly, the media were discarded, and the cells were rinsed with phosphate‐buffered saline (PBS) two times, followed by the treatment of pre‐warmed 0.5% TritonX‐100 in PBS at 37°C for 8 min. After the wash with PBS three times, the cells were frozen at −80°C for 4 h and thawed in pre‐heated PBS at 37°C for 40 min. Triplicate freeze/thaw (−80°C/37°C) cycles were performed. After that, the dECM samples were incubated in DNase (50 U/mL)/RNase (50 μg/mL) at 37°C for 2 h and rinsed with PBS three times. The dECM generated from MC3T3‐E1 was used to mimic bone microenvironment, which was defined as bone mimetic ECM (BM‐ECM). Meanwhile, the dECM generated from MG‐63 was used to mimic bone tumor microenvironment, which was defined as osteosarcoma ECM (OS‐ECM). Collagen coating was used as a negative control.

### 2.2 Residual DNA assessment

DAPI staining and residual DNA quantification were used to evaluate the residual DNA which was an important index for dECM. For DAPI staining, the samples were fixed with 4% formaldehyde for 15 min and rinsed with PBS. DAPI solution (#4083, Cell Signaling Technology) at the concentration of 1 μg/mL was added, and the samples were incubated for 5 min at room temperature avoiding light. After PBS washing, the samples were coated with anti‐fade mounting solution and imaged under a fluorescence microscope (Eclipse Ti2, Nikon).

The content of residual DNA was quantified by a cell proliferation assay kit (C7026, Invitrogen) according to the manual. Native cells were washed with PBS, frozen, and stored at −80°C. The frozen cells and dECMs were thawed at room temperature and incubated in CyQUANT® GR dye/cell‐lysis buffer for 2–5 min, protected from light. The samples were measured using a fluorescence microplate reader with filters for 480 nm excitation and 520 nm emission maxima. The standard curve was prepared with λDNA (provided by the kit). The DNA content of dECM divided by the DNA content of native cells was defined as relative DNA percentage.

### 2.3 Quantification of glycosaminoglycans and collagenous proteins in dECM

Glycosaminoglycan (GAG) content was assessed by GENMED GAG quantification kit via DMMB (CM51023.1, GENMED Scientifics Inc.). Briefly, dECM was scrapped mechanically from 24‐well plates and transferred into a 1.5‐mL Eppendorf (EP) tube. The samples were washed with GENMEDA solution, followed by centrifugation at 300 g/min for 15 min. The collected ECM was dissolved in GENMED B solution, undergone strong vortex shaking, and incubated in a water bath at 56°C for 16 h, followed by a further water bath at 96°C for 10 min. After that, 5 μL sample and 100 μL GENMED C were mixed with a vortex for 15 s and incubated at room temperature for 30 min in darkness. The samples were centrifuged at 16,000 g/min for 10 min, and the precipitation was vibrated vigorously with GENMED D. The mixed solution was transferred into a 96‐well plate and measured at the absorbance of 656 nm. The GAG content was calculated according to the standard curve prepared with standard GAG samples in the kit.

Collagen staining and quantification were performed by Sirius Red/Fast Green Collagen Staining Kit (9046, Chondrex), as previously reported.^28^, ^29^, ^30^ Briefly, dECM or cells in 24‐well plates were rinsed with PBS three times and fixed in Kahle fixative (26.7% ethanol/3.7% formaldehyde/2% glacial acetic acid in distilled water). After that, the samples were submerged in dye solution for 30 min at room temperature. Discarded dye solution and rinsed the sample surface with distilled water until the water was clear. The collagenous staining of the samples was observed under a microscope. Subsequently, 1 mL of dye extraction buffer was added, and the collagen content of the samples was calculated using the following formula based on the absorbance at 540 nm and 605 nm according to the manufacturer's instructions.

$$\text{Collagen}\;\left(\mathrm{\mu g}\;\mathrm{per}\;\text{well}\right)=\frac{\mathrm{OD}540\;\text{value}-\left(0.291\times \mathrm{OD}605\;\text{value}\right)}{0.0378}$$

### 2.4 Immunofluorescence staining

Immunofluorescence (IF) staining of fibronectin (FN), collagen type I alpha 1 (COL1A1), and F‐actin was performed as previously reported.^29^ Briefly, the samples were fixed with 4% formaldehyde solution for 15 min and rinsed with PBS. After that, the samples were penetrated with 0.3% TritonX‐100 in PBS for 10 min and rinsed with PBS, followed by blocking incubation (5% normal goat serum in PBS) for 1 h at room temperature. Primary antibodies were diluted in 1% BSA/PBS as below: FN (SC‐8422, Santa Cruz Biotechnology) (1:50), COL1A1 (ab21286, Abcam) (1:200), and F‐actin (FITC‐phalloidin) (CA1620, Solarbio) (1:100). After incubation with primary antibodies at 4°C overnight and PBS washing, secondary antibodies conjugated with Alexa Fluor® 555 or Alexa Fluor® 488 (Cell Signaling Technology) were applied except F‐actin stained samples. Imaging was caught under an inverted fluorescence microscope.

### 2.5 ECM stiffness testing

BM‐ECM‐ and OS‐ECM‐coated plates were prepared for mechanical characteristics. The empty plates and COLI‐coated plates were served as negative controls. Each sample was examined at least 50 different locations (each point 3 mm apart on the X or Y axis) by Piuma Nanoindenter (Optics11, The Netherlands) with a loading speed of 3 mm/s. Young's modulus was calculated accordingly by the instrument.

### 2.6 Cell adhesion on biomimetic ECM

Cell morphology and adhesion ratio were performed to investigate the adhesion activities of breast cancer cells on biomimetic dECM materials. Breast cancer cells were inoculated on 24‐well plates at the density of 5 × 10^4^ cells/well. At different time points (1, 2, 4, and 8 h), unattached cells were washed two times with PBS and incubated in cell counting solution (Cell proliferation kit, K1018, Ape×Bio) at 37°C for 2 h. The absorbance at 450 nm was measured. The adhesion ratio was calculated using the following formula, while the cell adhesion at 8 h was defined as 100%:

$$\text{Cell adhesion ratio}\;\left(\%\right)=\frac{\mathrm{OD}450\;\left(\text{desired time point}\right)-\mathrm{OD}450\;\left(8\;h\right)\;}{\mathrm{OD}450\;\left(8\;h\right)}$$

To assess the cell morphology of initial adhesion, breast cancer cells (5 × 10^4^ cells/well) were inoculated in 24‐well plates coated with different biomimetic dECMs. At different time points (1, 4, and 16 h), the cells were stained with FITC‐phalloidin to show cytoskeletal proteins (F‐actin). The experiments were performed according to the IF staining method described above. DAPI was used to show the nucleus. The cell morphology images were taken under the fluorescence microscope.

### 2.7 Cell migration on biomimetic ECM

Wound‐healing assay, transwell migration assay, and colony formation were performed to investigate the migration ability of breast cancer cells on the mimetic ECM stroma.

For wound‐healing assay, breast cancer cells were starved in basic medium with 5% FBS for 12 h. After that, the cells were trypsinized into single cells and cultured on a 12‐well plate coated with dECMs at the density of 1 × 10^5^ cells/well for another 12 h. The cells with the coated dECMs were scratched in the center of each well with a 200‐μL pipette tip. The dropped cells and dECMs were washed with PBS. The migrated cells were recorded by a microscope at desired time points. The left area of the scratches without cells was quantified using ImageJ. The cell migration rate at each time point was normalized by the initial scratch area.

For transwell migration assay, MC3T3 or MG63 cells (3 × 10^5^ cells/well) were pre‐inoculated on the bottom of the transwell chamber for 14 days. The posterior chamber containing biomimetic ECM at the sublayer was prepared by decellularization as described previously. Breast cancer cells (1 × 10^5^ cells/well) were seeded in the inserts. PRMI 1640 with low FBS (3%) was added in the top chamber of transwell, while PRMI 1640 with high FBS (10%) was added in the lower section. After an appropriate time of inoculation, the cells were fixed with 4% formaldehyde, and treated with 0.1% crystalline violet staining solution for 10 min. The upper surface cells were gently wiped off with sterile cotton swabs, air‐dried, and observed under a microscope. The images were taken randomly, and the area of crystalline violet was quantified using ImageJ.

Clonal island formation was introduced to assess the distance of newborn cells from original cells. Breast cancer cells (1 × 10^3^ cells/well) were singly seeded on 6‐well plates coated with biomimetic ECM and cultured for 7 days with fresh medium replaced every other day. Clonal islands of breast cancer cells were formed, imaged under a microscope, and measured the size. Clonal islands with close cell numbers were selected for quantification of clonal island diameter using ImageJ software.

### 2.8 RNA isolation and real‐time qPCR

Cells were rinsed with PBS and lysed in Omega RNA‐Solv® Reagent (R6830‐01). Total mRNA was extracted according to the manufacturer's instructions. The extracted mRNA was reverse transcribed to cDNA by One‐Step gDNA Removal and cDNA Synthesis Supermix (AT311, TRANSGEN). Real‐time qPCR was performed with TransStart® Top Green qPCR SuperMix (P41014, TRANSGEN) was used for qPCR.

CXCR4, VEGF, and BSP were typical genes associated with bone metastasis of breast cancer. E‐cadherin, N‐cadherin, and Vimentin were used as EMT markers. The primers of these genes were synthesized by Generay Biotech Co., Ltd. The primer sequences are shown in Table 1.

**TABLE 1:** Primer sequences for real‐time qPCR amplification.

Gene	Species	Primers (F = forward, R = reverse)
Bsp	Mouse	F: CAGAGGAGGCAAGCGTCACT
		R: CTGTCTGGGTGCCAACACTG
BSP	Human	F: CAACAGCACAGAGGCAGAAA
		R: CGTACTCCCCCTCGTATTCA
Cxcr4	Mouse	F: GACCGCCTTTACCCCGATAG
		R: CCCTTGGAGTGTGACAGCTT
CXCR4	Human	F: GGGCAATGGATTGGTCATCCT
		R: TGCAGCCTGTACTTGTCCG
Vegf	Mouse	F: GGCGAGGCAGCTTGAGTTA
		R: TCAACGGTGACGATGATGGC
VEGF	Human	F: AGGGCAGAATCATCACGAAG
		R: GCAGCAGCCCCCGCATCGCATCAGG
N‐cadherin	Mouse/human	F: GACAATGCCCCTCAAGTGTT
		R: CCATTAAGCCGAGTGATGGT
E‐cadherin	Mouse	F: CAGTTCCGAGGTCTACACCTT
		R: TGAATCGGGAGTCTTCCGAAAA
E‐cadherin	Human	F: TGAAGGTGACAGAGCCTCTGGAT
		R: TGGGTGAATTCGGGCTTGTT
Vimentin	Mouse/human	F: CCAAACTTTTCCTCCCTGAACC
		R: GTGATGCTGAGAAGTTTCGTTGA
β‐Actin	Mouse	F: AGATGTGGATCAGCAAGCAG
		R: GCGCAAGTTAGGTTTTGTCA
GAPDH	Human	F: GGTGGTCTCCTCTGACTTCAACA
		R: GTTGCTGTAGCCAAATTCGTTGT

### 2.9 ELISA assay

Breast cancer cells (5 × 10^5^ cells/well) were inoculated into six‐well plates coated with different biomimetic ECM and cultured for 4 days. Supernatants were collected in EP tubes and centrifuged at 3000*g*/min for 20 min. The collected supernatant was subjected to cell‐secreted VEGF content assay with VEGF ELISA assay kits (Proteintech Group, Inc.) depending on the cell species. Meanwhile, cell lysate was collected via trypsin digestion and centrifugation at 1000*g*/min for 5 min, followed by triplicate cycling at 20°C/−80°C. The cell lysate was subjected to intracellular BSP content assay with BSP ELISA kit (Animalunion Biotechnology) depending on the cell species. According to the manufacturer's instructions, the samples were added to a VEGF/BSP antibody‐coated microplate with horseradish peroxidase (HRP)‐labeled detection antibody, and the wells were sealed with a sealing membrane for 60 min at 37°C. Subsequently, the plate was washed five times with washing solution, and the substrate was added for 15 min at 37°C, protected from light. The absorbance was measured at 450 nm. The concentration of VEGF or BSP was calculated with a standard curve performed accordingly.

### 2.10 Protein extraction and western blotting analysis

To extract ECM proteins, the dECM was mechanically separated from the tissue culture plates and placed in UA buffer (8 M urea, 150 mM Tris–HCl, pH 8.0) at 37°C for 2 h. Subsequently, the samples were sonicated (80 W, 10 s on/15 s off, 10 cycles) and placed in boiling at 100°C for 30 min. ECM protein samples were obtained by centrifuging the samples at 14,000*g*/min for 40 min. To extract intercellular proteins, the cells were lysed in RIPA buffer (R0010, Solarbio) containing 1 mM PMSF at 4°C for 20 min. The lysates were collected in EP tubes and centrifuged (12,000 rpm/4°C/30 min). The supernatant was transferred to a new tube for protein quantification. A standard curve was prepared with gradient concentration of BSA according to the instructions of a BCA quantification kit. The total protein amount for ECM protein (COLI and FN) assay was 50 μg per well, while the total protein amount for other protein assay was 20 μg per well.

For western blotting assay, the protein samples were mixed with 5 × loading buffer and heated for 10 min at 98°C. The proteins were separated with SDS‐PAGE gel and transferred to a nitrocellulose filter membrane. The membrane was rinsed with TBS‐T, followed by Fast Blocking Solution (P30500, New Cell & Molecular Biotech Co., Ltd [NCM Biotech]) for 10 min. The samples were incubated in primary antibodies at 4°C overnight. The Vimentin antibody (A19607, Abclonal) was diluted at 1:5000 in antibody dilution solution (WB500D, NCM Biotech). E‐Cadherin antibody (20874‐1‐AP, Proteintech) was diluted at 1:1000. CXCR4 antibody (60042‐1‐LG, Proteintech) was diluted at 1:1000, and β‐actin (AC026, Abclonal) was diluted at 1:10,000. The samples were then incubated with HRP‐coupled secondary antibodies of the corresponding species (Sa00001‐2, AS003, Abclonal). Original blots were provided in the Supporting Information (Figures S1–S3).

### 2.11 Animal studies

The commercial small intestinal submucosa (SIS) scaffolds were cut into circular sizes with 5 mm diameter. The dried scaffolds were pretreated with PBS for at least 16 h and with α‐MEM medium for another 16 h. MC3T3‐E1 and MG63 cells were seeded on the scaffolds, respectively. The cells were cultured for 5 days in complete medium and for another 7 days with ascorbic acid (50 μg/mL). The ECM‐coated scaffolds were prepared following decellularization as described in Section 2.1. Female BALB/c nude mice at the age of 8–10 weeks were purchased from Charles River and housed under specific pathogen‐free conditions in the Animal Center of Ningbo University. Five days after arriving, the mice were randomly divided into three groups: (I) COLI‐SIS group (*n* = 5); (II) BM‐ECM‐SIS group (*n* = 6); and (III) OS‐ECM‐SIS group (*n* = 6). The dECM coating SIS prepared above was surgically implanted below the scapulae of the nude mice. Briefly, the nude mice were treated with 1% pentobarbital sodium (50 mg/kg) for anesthesia and small incisions were made at the dorsal part of the scapulae bilaterally using surgical scissors. The dECM‐coated SIS scaffolds were placed under the skin at the bilateral incisions, and the incisions were sewed up with sutures. The scaffolds were fixed on the skin to avoid displacement.

After 2 days, breast cancer cells were transplanted orthotopically in the fat pads of the right fourth mammary glands in nude mice. A surgical incision was made around, and 1 × 10^6^ 4T1‐RFP cells in PBS were injected into the fat pads. After 10 days of tumor cell implantation, the nude mice were captured under IVIS Lumina III (PerkinElmer) at excitation wavelength of 580 and absorption wavelength of 620 nm. Besides in vivo evaluation, all scaffolds were stripped from the nude mice and captured together to avoid the system operation error. The signals were quantified using Living Image software later. The volume and size of the primary tumors in mammary glands were measured.

Then, the scaffolds were fixed in 10% neutral buffered formalin fixative (G2162, Solarbio) overnight at room temperature. RFP‐labeled breast cancer cells on the scaffolds were assessed under confocal microscopy (SP8, LEICA). DAPI was used to show the nuclei.

All experimental procedures involving animals were conducted in compliance with Chinese legislation regarding the use and care of laboratory animals and were approved by the Animal Care and Use Committee, School of Medicine at Ningbo University. The approved protocol number is NBU20220099.

### 2.12 Statistical analysis

Statistical analyses were performed using the GraphPad Prism 8.0a (GraphPad Software). Data were presented as the mean ± SD. Student *t* test was used to determine statistical differences between the two groups. When more than two groups were analyzed, one‐way analysis of variance followed by a post hoc test (multi‐group comparison) was used. *p* < 0.05 was considered statistically significant.

## 3 RESULTS

### 3.1 Generation and characteristics of biomimetic dECMs

**FIGURE 1:**
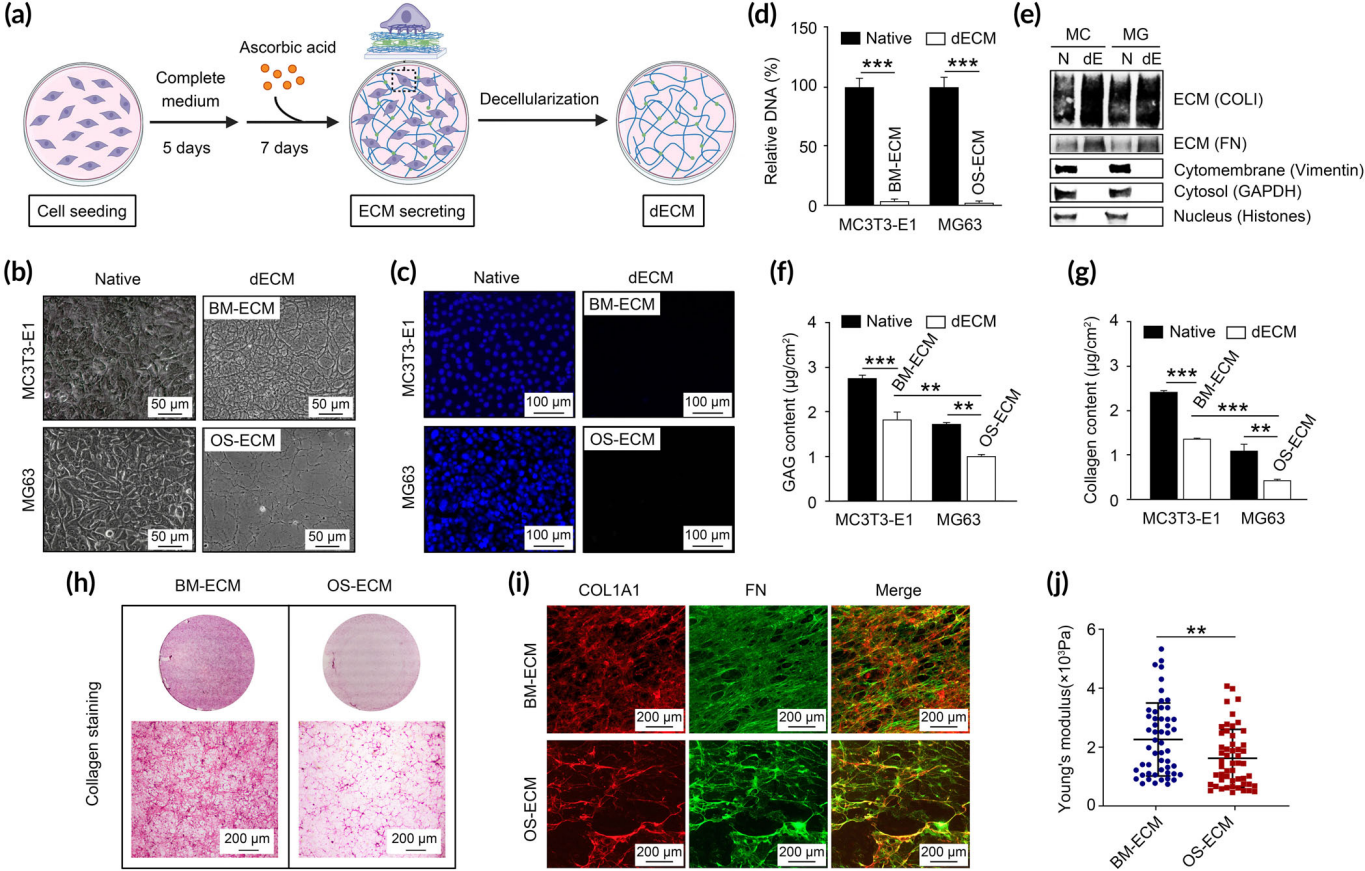
Bone mimetic ECM (BM‐ECM) exhibited higher levels of ECM components, denser structure, and higher stiffness compared to osteosarcoma mimetic ECM (OS‐ECM). (a) Schematic diagram of cell‐derived biomimetic ECM generation. (b) Bright‐field images taken by a phase contrast microscope before (Native) and after (dECM) decellularization. After decellularization, the cells were removed and ECM structure with interweaving network was observed. The dECM structure in BM‐ECM was denser than that in OS‐ECM. DAPI staining (c) and residual DNA quantification (d) revealed the removement of the cells via decellularization. The relative DNA (%) was defined as the DNA content in dECMs divided by that in the native cells before decellularization. (e) Western blotting of representative proteins in cells or ECMs. Cytomembrance protein (Vimentin), cytosol protein (GAPDH), and nuclear protein (Histones) were only expressed in native cells, indicating cellular components were removed via decellularization process. ECM proteins (COLI and FN) were enriched in dECMs, indicating ECM proteins were preserved after decellularization. MC: MC3T3‐E1, MG: MG63, N: native, dE: dECMs. (f) Quantification of GAG content. (G, H) The cells and dECMs were stained with Sirius Red/Fast Green Collagen Staining Kit. Collagen content was quantified according to the manufacturer's instructions of the kit (G). Collagen fibers were stained as red (H). (I) IF staining of COL1A1 (red) and OCN (green). (J) Young's modulus of biomimetic dECMs (BM‐ECM and OS‐ECM). At least 50 sites of each group were measured. **, *p* < 0.01; ***, *p* < 0.001. BM‐ECM, bone mimetic ECM; dECM, decellularized ECM; ECM, extracellular matrix; GAG, glycosaminoglycan; IF, immunofluorescence; OS‐ECM, osteosarcoma ECM.

The ECM microenvironment was specific to cell types and cell activities. Here, the dECM from preosteoblasts (MC3T3‐E1) were used to mimic normal bone microenvironment (BM‐ECM) which have been applied for bone regeneration in our previous studies.^27^ Meanwhile, the dECM from osteosarcoma cells (MG63) was used to mimic tumor microenvironment in bone tissue (OS‐ECM). The biomimetic dECMs were generated as shown in Figure 1a. Ascorbic acid was added to accumulate ECM secretion. Bright‐field images, DAPI staining, DNA content quantification, and western blotting were used to assess the effect of decellularization process (Figure 1b–e). The native cells were obviously removed, and the network structure of dECM was observed (Figure 1B). Compared to OS‐ECM, BM‐ECM was much more compact with higher binding affinity to tissue culture plates during decellularization. Residual DNA was less than 5% in dECMs compared to that in native cells (Figure 1c). After decellularization, the cell proteins in cytomembrane (Vimentin), cytosol (GAPDH), and nucleus (Histones) were almost cleared, while the ECM proteins (FN and COLI) were reserved and even slightly enriched (Figure 1e). The retained GAGs in BM‐ECM were 1.46 μg/cm^2^ (56.7%), which was significantly more than that in OS‐ECM (0.476 μg/cm^2^; 40.4%; Figure 1f). Similarly, compared to the OS‐ECM group, collagen content in BM‐ECM was much higher (Figure 1g), with heavier staining and denser structure as stained by collagen proteins (Figure 1h). IF staining of core ECM proteins (COL1A1 and FN) further confirmed the distinguished structure of the two kinds of biomimetic dECMs (Figure 1i). Consistently, BM‐ECM with compact ECM protein arrangement exhibited higher stiffness than OS‐ECM as shown by Young's modulus in Figure 1j. The Young's modulus of the empty plate and COLI‐coated plate was much higher than that in dECMs (Figure S4).

### 3.2 Distinguished adhesion activities of breast cancer cells assessed by the biomimetic ECM system

**FIGURE 2:**
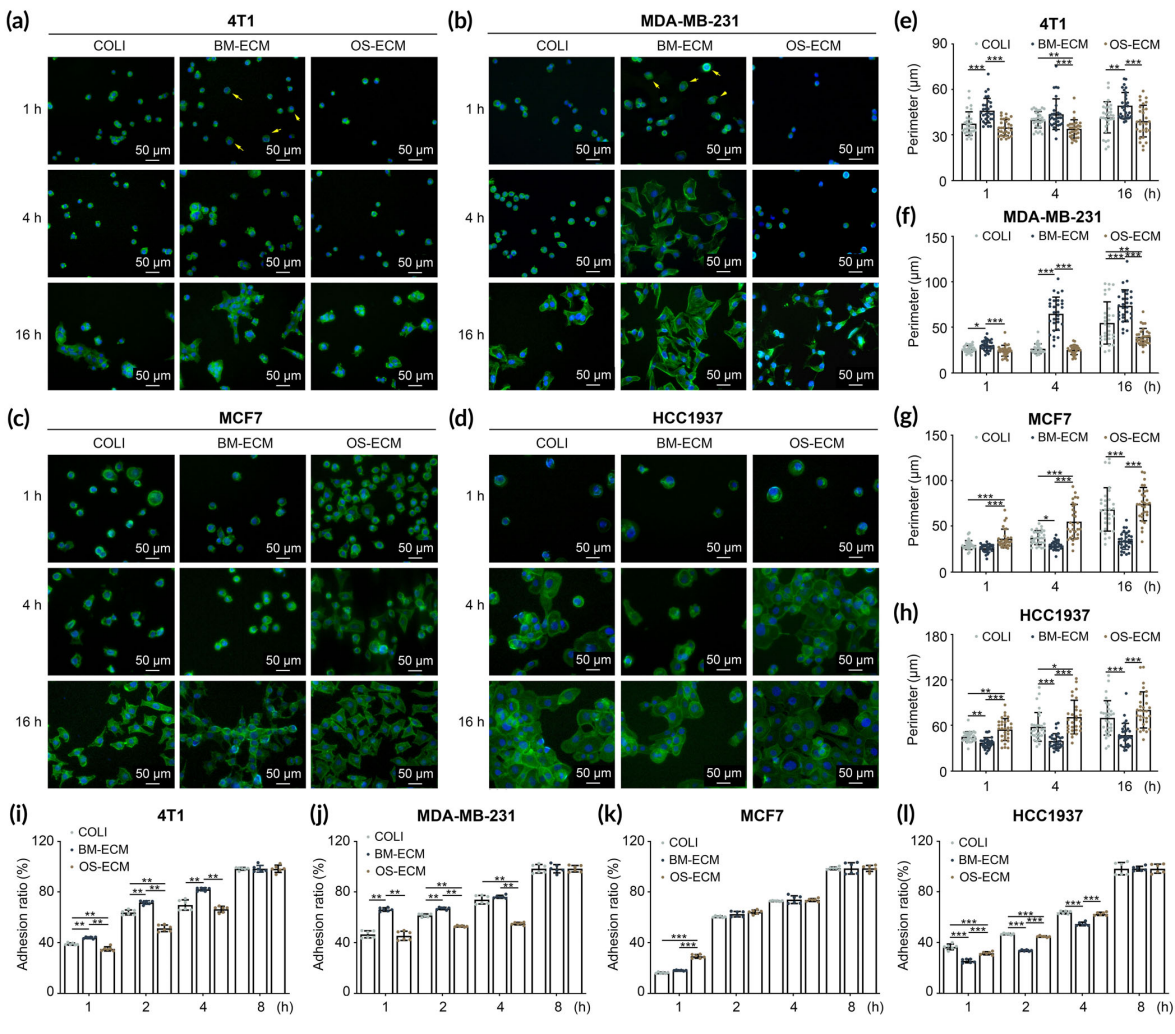
Cell adhesion assessment of breast cancer cells with different metastasis abilities on biomimetic dECMs (BM‐ECM and OS‐ECM). Breast cancer cells with high metastasis (4T1 and MDA‐MB‐231) and low metastasis (MCF7 and HCC1937) were seeded on BM‐ECM or OS‐ECM, respectively. COLI‐coated plates were introduced as a basic control, which was a major component in cell‐derived dECMs. (a–d) Representative images of attached cells to show the cell spreading morphology of 4T1 (a), MDA‐MB‐231 (b), MCF7 (c), and HCC1937 (d). F‐actin of the cells was stained by FITC‐phalloidin (green), and nuclei were stained by DAPI (blue). Better spreading cells on BM‐ECM at 1 h of 4T1 and MDA‐MB‐231 were pointed out with yellow arrows. (e–h) Quantification of cell perimeter by ImageJ software. At least 30 cells were measured for each group at each time point. (i–l) Quantification of cell adhesion ratio in different time points (1, 2, 4, and 8 h). The breast cancer cells (4T1 (i), MDA‐MB‐231 (j), MCF7 (k), and HCC1937 (l)) were seeded on COLI, BM‐ECM, and OS‐ECM for 8 h. Attached cell number was calculated with CCK kit at desired time point. Adhesion ratio was defined as the cell number at indicated times divided by the cell number at 8 h. Triplicate experiments were performed for each group. *, *p* < 0.05; **, *p* < 0.01; ***, *p* < 0.001. BM‐ECM, bone mimetic ECM; dECM, decellularized ECM; ECM, extracellular matrix; OS‐ECM, osteosarcoma ECM.

Breast cancer cells with different abilities of metastasis were seeded on BM‐ECM or OS‐ECM to investigate the affinity of biomimetic dECMs on cell adhesion associated with bone metastasis (Figure 2). The morphology of cell adhesion was assessed by F‐actin staining at different time points (1, 4, and 16 h). It is interesting to find that highly metastatic breast cancer cells (4T1 and MDA‐MB‐231) spread better on BM‐ECM than on OS‐ECM as early as 1 h (Figure 2a,b). The perimeter of 4T1 cells was the highest on BM‐ECM at all time points, compared to the other two groups, and statistical significance was observed between BM‐ECM and OS‐ECM (Figure 2e). The perimeter of MDA‐MB‐231 cells showed the same tendency as 4T1 cells (Figure 2f). In contrast, lowly metastatic breast cancer cells (MCF7 and HCC1937) tended to spread better with longer perimeter on OS‐ECM than on BM‐ECM (images: Figure 2c,d; quantification of perimeter: Figure 2g,h). Moreover, highly metastatic breast cancer cells were attached quicker on BM‐ECM with higher adhesion ratio than on OS‐ECM (Figure 2i,j), while the result was opposite in lowly metastatic breast cancer cells (Figure 2k,l). The results indicated highly metastatic breast cancer cells tended to adhere on bone mimetic microenvironment, while lowly metastatic breast cancer cells tended to adhere on bone tumor mimetic microenvironment.

### 3.3 Distinguished migration activities of breast cancer cells assessed by the biomimetic ECM system

**FIGURE 3:**
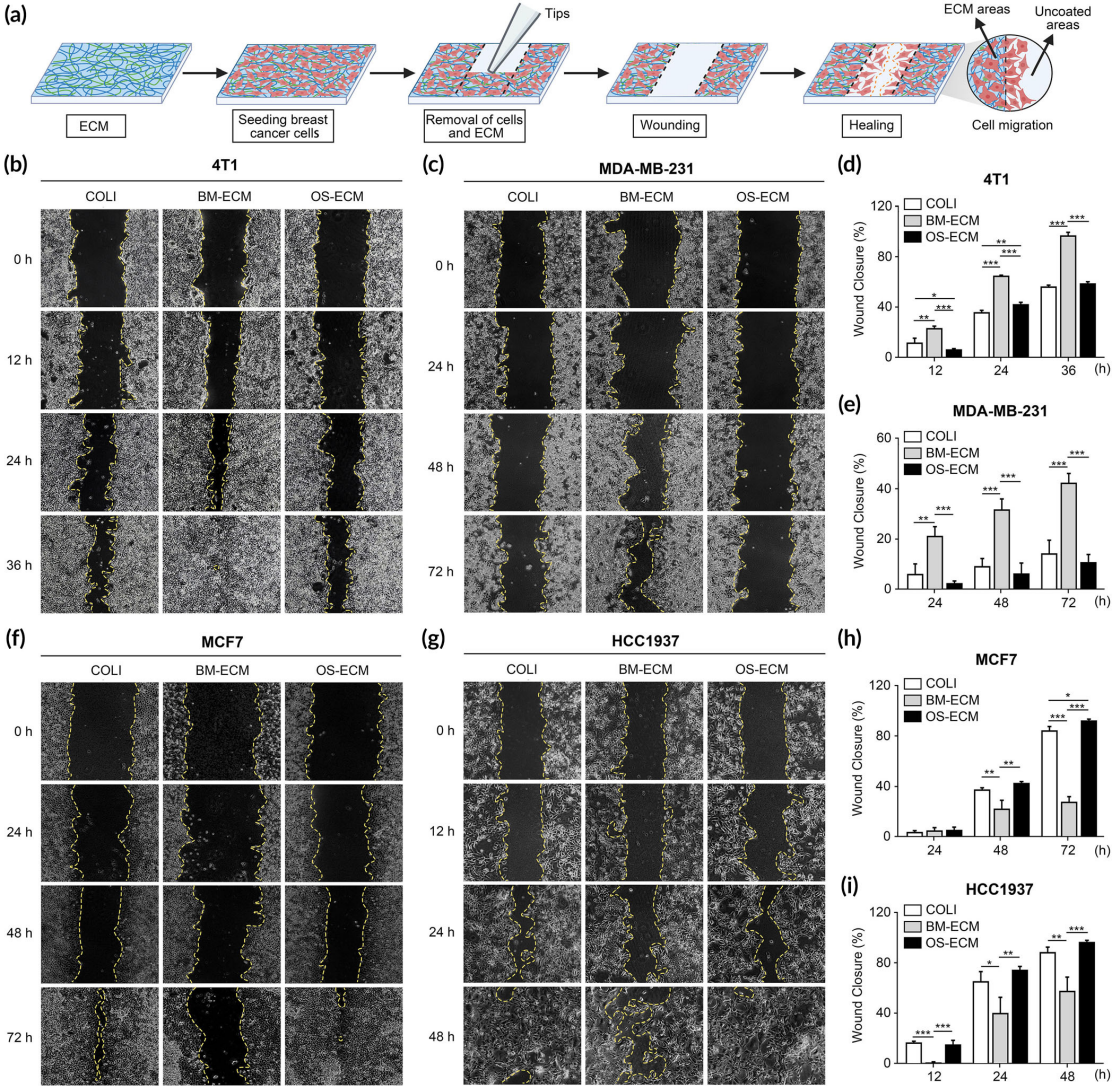
Cell migration assessment of breast cancer cells on biomimetic dECMs. Cells were seeded on COLI‐, BM‐ECM‐, and OS‐ECM‐coated plates. (A) Schematic of wound healing assay to assess the cell migration on ECM. A scratch was made with a 200‐μL pipette tip and washed with PBS. The cells with dECMs were removed from the scratched area. (b–e) Cell migration of highly metastatic breast cancer cells (4T1: b and d; MDA‐MB‐231: c and e). (f–i) Cell migration of lowly metastatic breast cancer cells (MCF7: f and h; HCC1937: g and i). Representative images of wound healing at different time points (b and c, f and g). Quantification of wound closure based on images by ImageJ software (d and e, h and i). The left area of scratch was displayed with yellow dotted line. The wound closure ratio was calculated with the migration area divided by the initial wound area. Triplicate experiments were performed for each group. *, *p* < 0.05; **, *p* < 0.01; ***, *p* < 0.001. BM‐ECM, bone mimetic ECM; dECM, decellularized ECM; ECM, extracellular matrix; OS‐ECM, osteosarcoma ECM; PBS, phosphate‐buffered saline.

**FIGURE 4:**
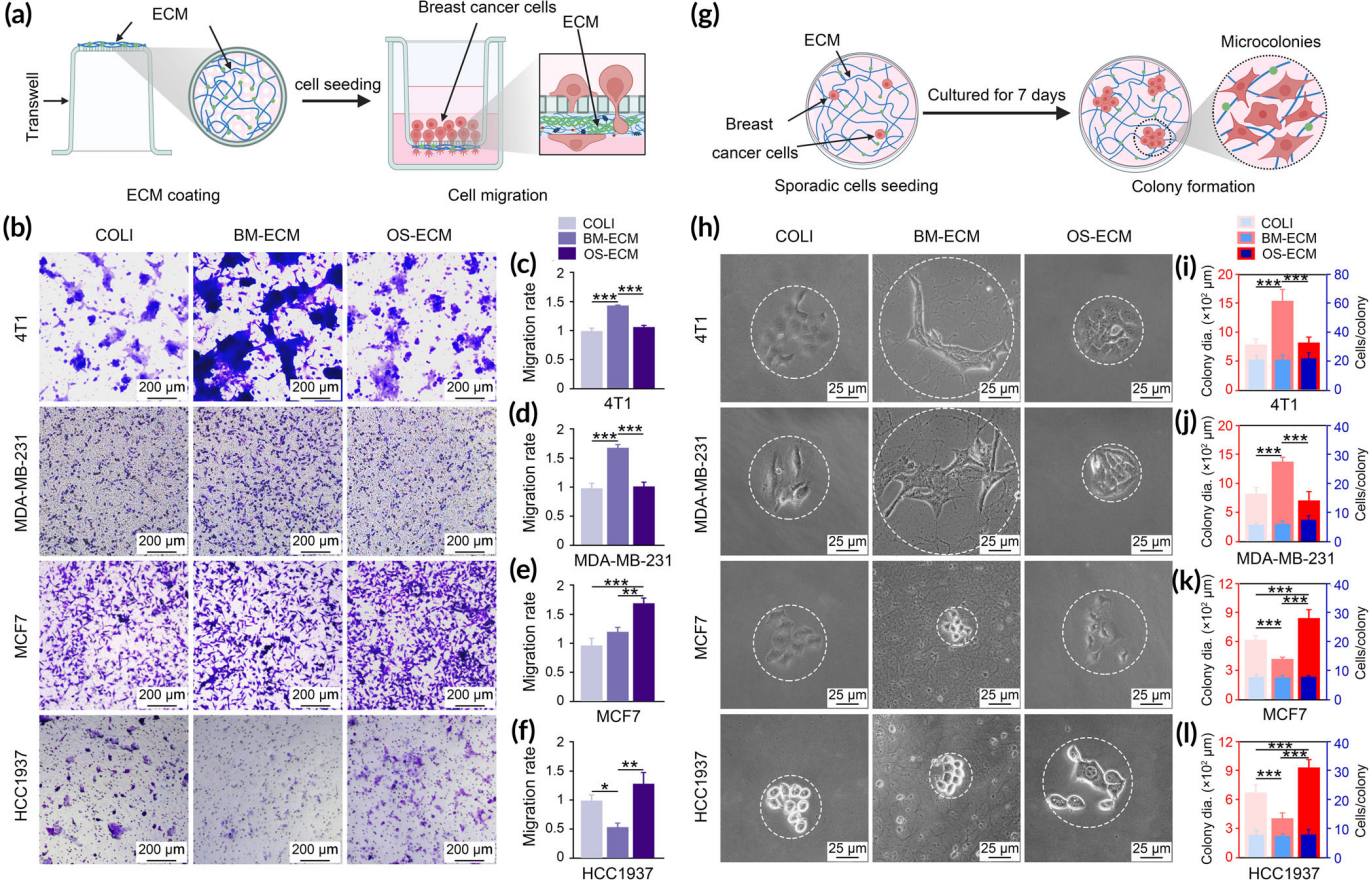
Migration of breast cancer cells with different metastatic abilities to biomimetic dECMs via transwell assay (a–f) and microcolony assay (g–l). (a) Schematic of breast cancer cell migration from unECM microenvironment to ECM microenvironment to evaluate the cell recruitment of dECMs. MC3T3‐E1 or MG63 cells were cultured on the lower layer of the transwell inserts for 10 days (5 days in complete culture medium and 5 days in the medium supplemented with 50 μg/mL ascorbic acid), followed by decellularization. Breast cancer cells were cultured on the upper layer of the filter membrane and migrated to the lower layer through the holes in the membrane under the attraction of dECMs. (b) Representative images of different breast cancer cells on dECMs. Migrated cells on the lower layer were stained with crystal violet as shown in purple. (c–f) Quantification of migrated cells on dECM‐coated membrane (4T1 (c), MDA‐MB‐231 (d), MCF7 (e), and HCC1937 (f)). The images were taken randomly, and the area of crystalline violet was quantified using ImageJ. At least nine images were used for quantification. (g) Schematic of microcolony assay. Breast cancer cells were singly seeded on dECM‐coated plates and cultured for 7 days. Clonal islands were formed. (h) Representative images of microcolonies formed by different breast cancer cells on dECMs. White dotted circles depicted the margins of the colonies. (i–l) Colony diameter and number of cells per colony of the breast cancer cells (4T1 (i), MDA‐MB‐231 (j), MCF7 (k), and HCC1937 (l)) on dECMs. At least five colonies were measured for each group. Triplicate experiments were performed for each group. *, *p* < 0.05; **, *p* < 0.01; ***, *p* < 0.001. dECM, decellularized ECM; ECM, extracellular matrix.

To determine the impact of ECM microenvironment on migration of breast cancer cells, wound healing (Figure 3) and transwell assay (Figure 4) were performed. Wound healing was used to evaluate the migration of cells on dECMs (Figure 3a), while transwell assay was used to evaluate the recruitment of cells migrated to dECM (Figure 4a–f). The breast cancer cells with different metastasis abilities were seeded on ECM‐coated plates (COLI, BM‐ECM, and OS‐ECM), and starved in medium with low serum to avoid the effect of cell proliferation. The results revealed that 4T1 and MDA‐MB‐231 cells migrated faster on BM‐ECM than on OS‐ECM or COLI control (Figure 3b,c), with the highest percentage of wound closure (Figure 3d,e). Reversely, MCF7 and HCC1937 cells migrated slowest on BM‐ECM (Figure 3f,g), with the lowest percentage of wound closure (Figure 3h,i).

For transwell assay, the biomimetic dECMs (BM‐ECM and OS‐ECM) were ornamented on the lower layer of the transwell insert membrane, while the breast cancer cells were seeded on the top layer of the transwell insert membrane as illustrated in Figure 4a. Under the guidance of the lower dECMs, breast cancer cells migrated through the holes in the insert membrane to the lower layer. Consistent with wound healing assay, highly metastatic breast cancer cells (4T1 and MDA‐MB‐231) preferred to migrate to BM‐ECM, while lowly metastatic breast cancer cells (MCF7 and HCC1937) preferred to migrate to OS‐ECM (Figure 4b). Quantification of the migration rate based on the crystal violet dyeing further confirmed the tendentiousness of the cells on different biomimetic dECMs (Figure 4c–f). For 4T1 and MDA‐MB‐231, the cell number migrated to BM‐ECM was significantly higher than that migrated to OS‐ECM (Figure 4c,d). On the contrary, for MCF7 and HCC1937, the cell number migrated to OS‐ECM was significantly higher than that to BM‐ECM (Figure 4e,f).

To further determine the effect of biomimetic dECMs on cell morphology and growth pattern, an alternate approach of microcolony formation was adopted as reported previously (Figure 4g).^31^ For 4T1, the cells grew tightly together on COLI and OS‐ECM. However, on BM‐ECM, the cells exhibited irregular cell morphology with numerous membrane protrusions (Figure 4h). Moreover, the colony size with similar cell numbers on BM‐ECM was significantly higher than on OS‐ECM (Figure 4i), which indicated the boosted migratory tendency of newborn cells from the original cells.^32^ A similar trend was observed in MDA‐MB‐231 cells (Figure 4h,j). Oppositely, MCF7 and HCC1937 cells were close together with round shape on BM‐ECM, while the cells were much looser and more polygonal on OS‐ECM (Figure 4h). Increase scattering of cells within the colony on OS‐ECM of such cells was corroborated by increase in colony size without significant increase in number of cells per colony (Figure 4k,l). The colony size of MCF7 and HCC1937 on BM‐ECM with similar cell number was the lowest among the groups (Figure 4k,l).

### 3.4 The expression of metastatic‐associated genes on biomimetic dECMs

**FIGURE 5:**
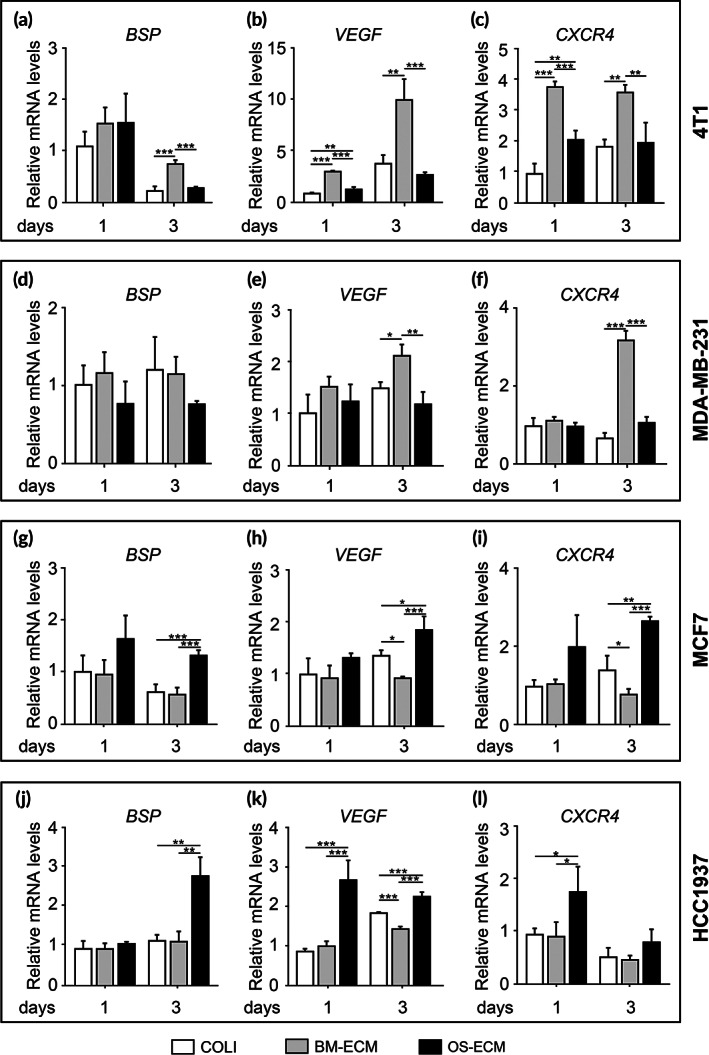
The effect of biomimetic dECMs on mRNA levels of metastatic‐related genes in breast cancer cells with different metastatic abilities. Breast cancer cells were cultured on COLI, BM‐ECM, and OS‐ECM for 3 days, respectively. The mRNA expression of metastatic marker genes (*BSP* (a, d, g, and j), *VEGF* (b, e, h, and k), and *CXCR4* (c, f, i, and l)) on Days 1 and 3 were assessed by quantitative real‐time PCR (qRT‐PCR). Both highly metastatic breast cancer cells (4T1 (a–c); MDA‐MB‐231 (d–f)) and lowly metastatic breast cancer cells (MCF7 (g–i) and HCC1937 (j–l)) were examined. Triplicate experiments were performed for each group. *, *p* < 0.05; **, *p* < 0.01; ***, *p* < 0.001. BM‐ECM, bone mimetic ECM; dECM, decellularized ECM; ECM, extracellular matrix; OS‐ECM, osteosarcoma ECM.

**FIGURE 6:**
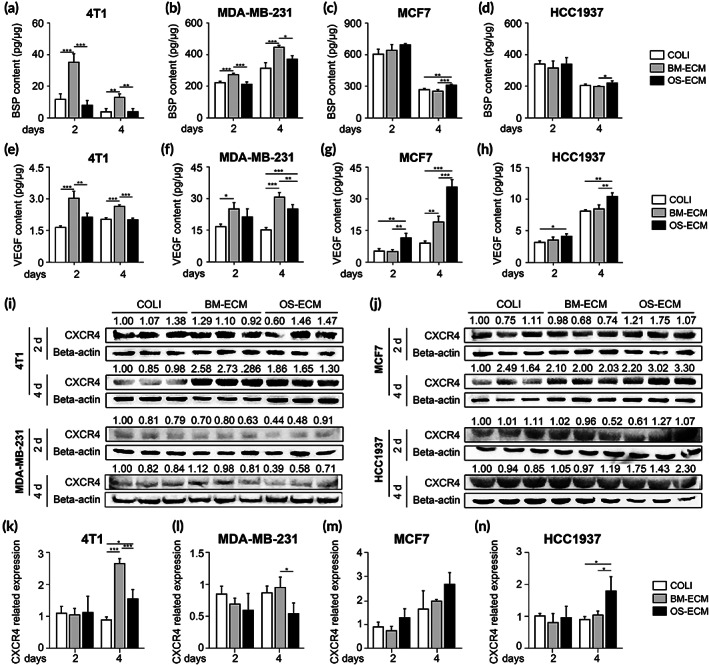
The effect of biomimetic dECMs on protein levels of metastatic‐related genes in breast cancer cells with different metastatic abilities. (a–h) The contents of BSP (a–d) and VEGF (e–h) were assayed by ELISA. Breast cancer cells with high metastasis ((4T1 (a and e), MDA‐MB‐231 (b and f)) and low metastasis (MCF7 (c and g), HCC1937 (d and h)) on dECMs at different time points (Days 2 and 4) were examined. (i–n) The protein levels of CXCR4 were assayed by western blotting in metastatic breast cancer cells (4T1 and MDA‐MB‐231) (i, k, and l) and low metastatic breast cancer cells (MCF7 and HCC1937) (j, m, and n) on dECMs at different time points (Days 2 and 4). (i and j) The WB bands were shown. Beta‐actin expression was served as an internal control. (k–n) Relative protein levels of CXCR4 were assessed by the quantification of the bands in (i and j) via ImageJ. Triplicate experiments were performed for each group. *, *p* < 0.05; **, *p* < 0.01; ***, *p* < 0.001. dECM, decellularized extracellular matrix.

Besides cell activities on the two kinds of biomimetic dECMs, the molecular levels of metastatic‐associated genes were further determined (Figures 5 and 6). To elaborate the metastatic activities induced by dECM niches from different perspective, bone sialoprotein (BSP), vascular endothelial growth factor (VEGF), and chemokine C‐X‐C motif receptor 4 (CXCR4) were chosen for assessment. BSP was an essential differentiation marker for osteogenesis. VEGF was a cytokine associated with angiogenesis, and CXCR4 was a critical chemokine receptor of CXCL12. All three genes have been reported to be highly expressed in breast cancer bone metastatic cells.^33^, ^34^, ^35^, ^36^ Here, the mRNA levels of BSP were the highest in 4T1 cells on BM‐ECM on Day 3, but no significant differences were found on Day 1 (Figure 5a). Besides that, the mRNA levels of both *VEGF* and *CXCR4* were the highest in 4T1 cells on BM‐ECM on Days 1 and 3 (Figure 5b,c). Similarly, the regulation of *VEGF* and *CXCR4* mRNA levels by biomimetic dECMs in MDA‐MB‐231 was more obvious than that of BSP, with the highest mRNA levels of *VEGF* and *CXCR4* in the cells on BM‐ECM on Day 3 (Figure 5d–f). Reversely, for MCF7 and HCC1937, the mRNA expression of the three genes was mainly highest in the cells on OS‐ECM on Day 3 (Figure 5g–l), except *CXCR4* expression in HCC1937 (Figure 5l). The regulation of *CXCR4* mRNA levels in HCC1937 with the significant difference occurred as early as Day 1 (Figure 5l).

The protein levels of BSP, VEGF, and CXCR4 in the cells on different biomimetic dECMs were further determined (Figure 6). BSP and VEGF protein expression was quantified by ELISA kit, and CXCR4 protein expression was measured by western blotting. In general, the protein expression tendency was basically consistent with that of mRNA. In detail, the protein levels of BSP were significantly up‐regulated in 4T1 and MDA‐MB‐231 cells on BM‐ECM on Days 2 and 4 (Figure 6a,b), which was more significant than the mRNA levels (Figure 5a,d). In contrast, BSP was highly expressed in MCF7 and HCC1937 cells on OS‐ECM (Figure 6c,d). Similar to BSP, VEGF proteins were expressed highest in 4T1 and MDA‐MB‐231 cells on BM‐ECM (Figure 6e,f), while the expression was the highest in MCF7 and HCC1937 cells on OS‐ECM on Days 2 and 4 (Figure 6g,h). The regulation of CXCR4 protein expression by biomimetic dECMs mainly occurred on Day 4 with the similar tendency (Figure 6i,j). For 4T1 and MDA‐MB‐231 cells, the highest levels of CXCR4 proteins were observed in the BM‐ECM group (Figure 6i). Meanwhile, for MCF7 and HCC1937 cells, CXCR4 protein expression was the highest in the OS‐ECM group (Figure 6j). Besides that, TFF1 expression was demonstrated to be associated significantly with bone relapse of breast cancer patients, with the highest‐ranking gene in bone metastatic breast tumors.^37^ Consistently, TFF1 was significantly up‐regulated in 4T1 cells on BM‐ECM, while it was significantly up‐regulated in MCF7 cells on OS‐ECM (Figure S5).

### 3.5 The promotion of cell migration by BM‐ECM was initiated with EMT

**FIGURE 7:**
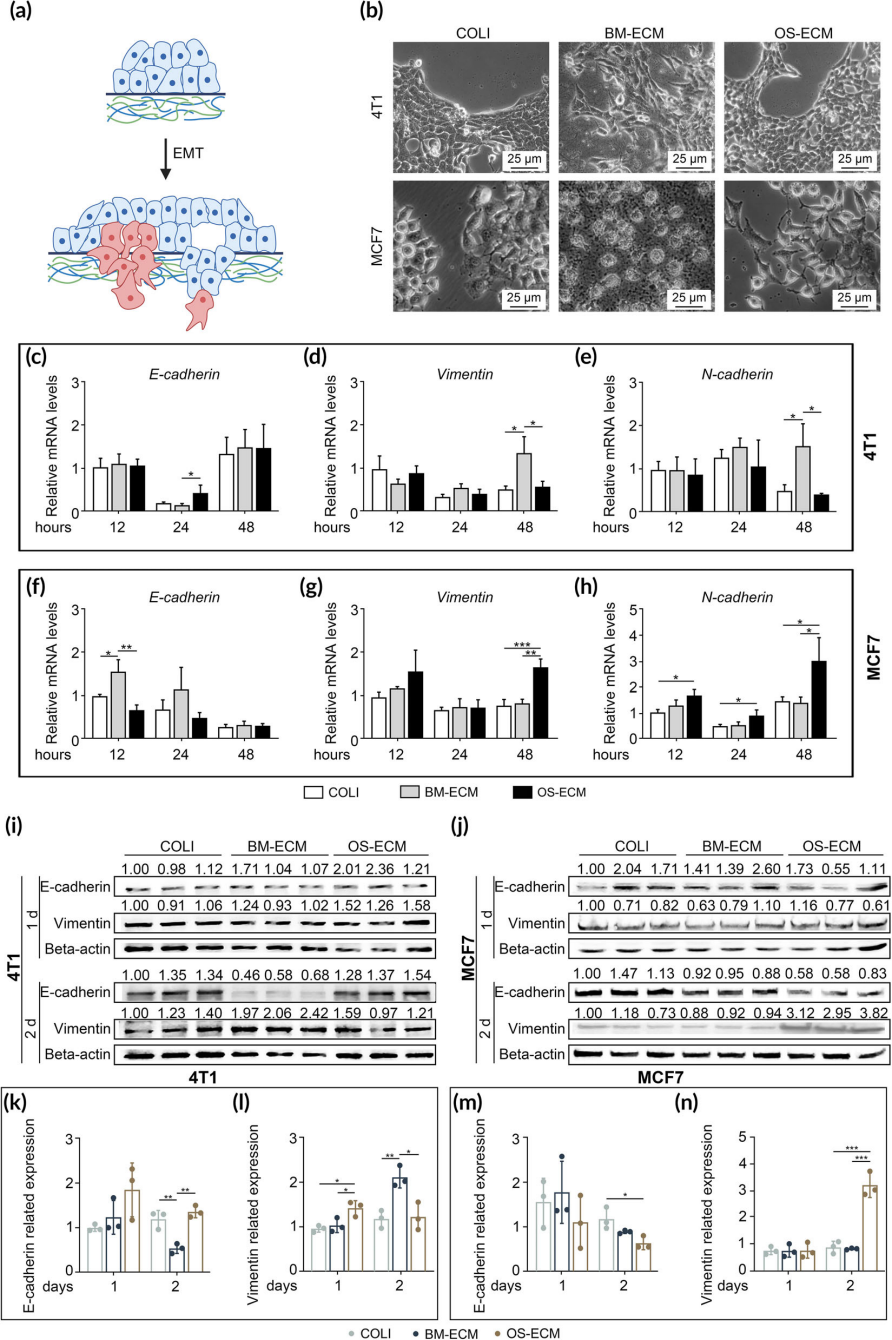
Effect of biomimetic dECMs on the phenotypic transition of breast cancer cells during EMT process. (a) Schematic diagram to show EMT progression of breast cancer cells on dECMs. (b) Representative images of cell morphology. Metastatic (4T1) and low metastatic (MCF7) breast cancer cells were cultured for 48 h on COLI, BM‐ECM, or OS‐ECM, respectively. Cell morphology was observed under phase contrast microscopy. (c–h) The mRNA expression of EMT‐related genes as measured by real‐time PCR. The breast cancer cells with high metastasis (4T1 (c–e)) and low metastasis (MCF7 (f and g)) on dECMs were assayed at different time points (12, 24, and 48 h). E‐cadherin (c and f), vimentin (d and g), and N‐cadherin (e and h) expression were assessed. (i–n) The protein expression of EMT‐related genes as measured by western blotting. (i and j) The WB bands were shown. Beta‐actin expression was served as an internal control. The bands in (i and j) were quantified via ImageJ (k–n). The purpose protein value divided by the β‐actin value was used to calculate the relative protein expression. E‐cadherin and Vimentin expression were assayed in metastatic breast cancer cells (4T1 (i, k, and l)) and low metastatic breast cancer cells (MCF7 (j, m, and n)) on dECMs at different time points (Days 1 and 2). Triplicate experiments were performed for each group. *, *p* < 0.05; **, *p* < 0.01; ***, *p* < 0.001. BM‐ECM, bone mimetic ECM; dECM, decellularized ECM; ECM, extracellular matrix; EMT, epithelial‐to‐mesenchymal transition; OS‐ECM, osteosarcoma ECM.

The epithelial‐to‐mesenchymal transition (EMT) has been proposed to contribute to the metastatic spread of breast cancer cells.^38^ Thus, EMT might be a potential mechanism of cell migration. To identify the hypothesis, the cell morphology and typical EMT‐associated markers (E‐cadherin, Vimentin, and N‐cadherin) were monitored (Figure 7). metastatic breast cancer cell 4T1 and low metastatic breast cancer cell MCF7 were used to assess the EMT process on biomimetic dECMs (Figure 7a). As shown in Figure 7b, 4T1 cells cultured on COLI and OS‐ECM were grown as typical island with clear and smooth edges, while partial cells on BM‐ECM became as long fusiform and escaped from the “island.” Though the cells on BM‐ECM seem significantly granular, live/dead staining confirmed the viability of MCF7 cells (Figure S6). Consistent with the cell morphology, the expression of E‐cadherin was lower in 4T1 cells on BM‐ECM than on OS‐ECM at mRNA levels after 24 h (Figure 7c) and at protein levels on Day 2 (Figure 7i). Meanwhile, the expression of mesenchymal markers (*Vimentin* and *N‐cadherin*) was significantly higher in 4T1 cells on BM‐ECM than the other two groups at mRNA levels after 48 h (Figure 7d,e). Vimentin protein was also expressed highest in 4T1 cells on BM‐ECM (Figure 7i). Oppositely, for MCF7 cells, partial cells on OS‐ECM exhibited long shuttle shape, while the cells on BM‐ECM were rounder (Figure 7b). Though the cells on BM‐ECM were separated from each other which might be caused by the cell proliferation inhibition, the interaction between cells and matrix was enhanced. E‐cadherin was expressed highest in the BM‐ECM group at mRNA levels after 12 h (Figure 7F), while the mesenchymal markers (*Vimentin* and *N‐cadherin*) were expressed highest in the OS‐ECM group at mRNA levels after 48 h (Figure 7g,h). The upregulation of E‐cadherin in the BM‐ECM group was observed at protein levels on Day 2, compared to the COLI and OS‐ECM groups (Figure 7j). Besides the phenotype‐associated genes, the essential transcription factors (TFs) (SNAI1, SLUG, and TWIST1) during EMT process were further assayed at mRNA levels (Figure S7). Consistently, the expression of the EMT‐TFs was significantly higher in 4T1 cells on the BM‐ECM than the other two groups at mRNA levels, while they were upregulated on the OS‐ECM in MCF7 cells, compared to BM‐ECM and COLI. Taken together, BM‐ECM promoted EMT of 4T1 cells, while OS‐ECM promoted EMT of MCF7 cells, which might contribute to the cell migration of breast cancer cells on different biomimetic dECMs.

### 3.6 Early detection of metastatic breast cancer cells in vivo

**FIGURE 8:**
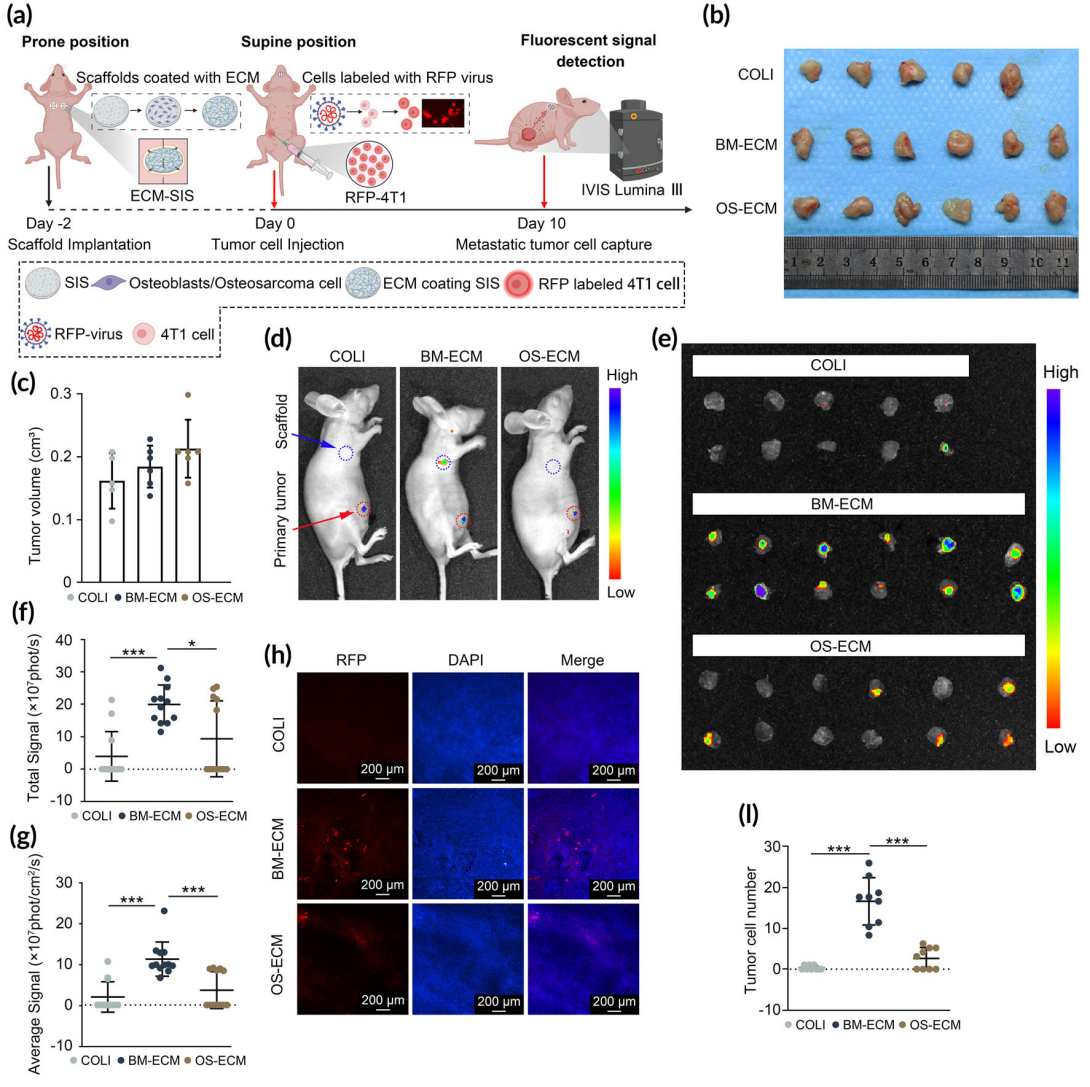
BM‐ECM enabled early capture of metastatic breast cancer cells in nude mice, compared to OS‐ECM. (a) Schematic of the in vivo model for the early detection of metastatic breast cancer cells. SIS scaffolds were coated with COLI, BM‐ECM, or OS‐ECM, followed by the implantation below the scapulae of the nude mice. After 2 days, RFP‐labeled 4T1 cells were injected into the right fourth mammary gland. On the 10th day after injection, the nude mice were captured under IVIS Lumina. (b) The primary tumors were stripped and pictures taken together (*n* = 5 for COLI group; *n* = 6 for BM‐ECM and OS‐ECM groups). A steel ruler was beside to show the size of the tumors. (c) Quantification of the tumor volume. (d) Representative images of the nude mice to show the signals of primary tumors and ECM‐coated scaffolds in vivo. Blue dotted circle: ECM‐SIS scaffolds; red dotted circle: primary tumor in mammary gland. (e) The scaffolds were striped and captured together (*n* = 10 for COLI group; *n* = 12 for BM‐ECM and OS‐ECM groups). The total signals (f) and average signals (g) on the scaffolds were calculated with Living Image software. (h) The striped scaffolds were captured under a confocal microscope. DAPI was stained to show the nuclei (blue). RFP‐4T1 cells were shown as red. (i) Quantification of tumor cells recruited on the ECM‐coated scaffolds. At least nine images from random fields were captured for each scaffold. *, *p* < 0.05; ***, *p* < 0.001. BM‐ECM, bone mimetic ECM; ECM, extracellular matrix; OS‐ECM, osteosarcoma ECM; SIS, small intestinal submucosa.

Based on the above results, we have found an interesting phenomenon. The cell activities of breast cancer cells on different biomimetic dECMs were highly associated with their metastasis abilities. Metastasis breast cancer cells were more inclined to adhere and migrate on the normal bone matrix microenvironment. However, low metastasis breast cancer cells preferred to adhere and migrate on the tumor matrix microenvironment. The association between them might be conductive to the early diagnosis of breast cancer metastasis. To demonstrate the hypothesis, we designed an in vivo model with implantation of biomimetic dECM‐coated scaffolds and metastatic breast cancer cells, as illustrated in Figure 8a. Whether the biomimetic ECM system can successfully capture the early metastatic breast cancer cells was assessed later. After injection of RFP‐4T1 cells for 10 days, obvious primary tumors were found in the right fourth mammary glands (Figure 8b). The tumor size was later quantified, and no significant difference was detected among different groups (Figure 8c). Under the luminescence scan, the strongest signal was observed in the primary tumor in the right fourth mammary gland (Figure 8d, red dotted circle). Meanwhile, obvious signal could be found in the BM‐ECM scaffolds, but almost no signal could be found in the other two groups (Figure 8d, blue dotted circle). To make more comparable and precise, the scaffolds with different ECM coatings were stripped from the mice and scanned together (Figure 8e). Consistently, the fluorescent signals were at high level in the BM‐ECM group, while negative signal was observed in the COLI group and slight signals were observed in the OS‐ECM group. The quantification of total signals and average signals revealed the highest level in the BM‐ECM group significantly, compared to the other two groups (Figure 8f,g). Moreover, no positive signal was observed in the potential metastatic organs (heart, liver, spleen, lung, kidney, and bone), while significant signals were observed in BM‐ECM scaffolds at the same time (Figure S8). The results indicated that such biomimetic dECM system might be a promising tumor model for the early detection of metastatic breast cancer cells.

To further confirm the recruitment of RFP‐labeled tumor cells, the ECM‐coated scaffolds were fixed, stained with DAPI, and captured under a confocal microscope (Figure 8h). A similar trend was observed with more RFP‐labeled tumor cells on the BM‐ECM scaffolds than in the other two groups. Statistical significance was assessed (Figure 8i).

## 4 DISCUSSION

The microenvironment of target organs was essential for tumor metastasis via cell‐matrix interactions. Bone was the major metastatic site of breast cancer, which indicated the bone niche was suitable for breast cancer cells colonization. Based on this, the bone matrix might harbor different ECM proteins under physiological or pathological conditions and further affect tumor cell activities, including cell adhesion and migration. Here, we developed two kinds of dECM to mimic bone matrix microenvironment under different situations. Decellularized ECM from normal preosteoblasts (BM‐ECM) was used to mimic the normal bone matrix, while dECM from osteosarcoma cells was used to mimic the bone tumor matrix. The results demonstrated that comparative analysis of cell activities on both was helpful to distinguish the bone metastasis ability of breast cancer cells in vitro and facilitated early detection of metastatic breast cancer cells in vivo. Therefore, the two kinds of dECM could be taken together as a biomimetic ECM system to be applied in breast cancer bone metastasis.

Previously, synthetic biomaterials were introduced to capture early metastatic breast cancer cells. For example, poly (lactide‐co‐glycolide) (PLG) scaffolds^39^ and micro‐porous poly (ε‐caprolactone) (PCL) scaffolds^8^ were fabricated and successfully for metastatic cells capture in vivo, which achieved high cell densities and reduced the tumor burden within solid organs. The scaffold‐captured tumor cells were further demonstrated to be similar to those at metastatic sites.^40^ Though such chemosynthetic scaffolds were proved to be nontoxic, their biological activity and biomimetic performance are still limited, lacking support from biological macromolecules. In contrast, ECM was considered as a competitive biomaterial with appropriate niches. Abundant research proved biomimetic supportive network with a plethora of extracellular signaling molecules. ECM‐based biomaterials have attracted plenty of attentions as smart platforms for dynamic functionality, such as cell traction, cell‐ECM interactions, and cell‐mediated ECM remodeling.^41^ Initially, ECM proteins (COLI, COLIV, and fibronectin) were used to decorate synthetic scaffolds to increase the cell adhesion and proliferation activity. Even though simply mixture of several ECM components was still hard to mimic the complex structure of ECM with abundant biomolecules as the native microenvironment to monitor the colonization process in vivo.

During the last decades, dECM was of particular interest to provide a microenvironment close to the native target tissue.^42^ Originally, tissue‐derived dECM was widely applied for total organ or tissue replacement and regeneration with excellent biocompatibility. However, material source, vigorous decellularization process to diminish the structural stability and potential problems still limited its application. Nowadays, cell‐derived ECM has gradually entered researchers' vision with some advantages. For example, most required cells were easy to obtain and expand. The decellularization process of cells was much gentler than tissues to preserve the precise ECM structure and component distribution. Most importantly, cell‐derived ECM was more selectivity and control lability since cell types and culture conditions and exhibited distinguished cell activities. Our previous studies focused on cell‐derived dECM and developed a serial of bone mimetic dECMs via alteration of culture situation, including osteogenic induction,^43^ mineralization by calcium,^16^ and fibroblasts‐osteoblasts coculture.^28^ In these studies, the morphology, expression, and distribution of ECM core matrisome proteins (COLI and FN) showed obvious differences, which determined variant cell fate. Furthermore, increasing evidence demonstrated the differences in ECM protein composition and proportion among different cell types or under different conditions of the same cell via proteomics. Soucy et al. reported that the major components in the ECM from human fetal lung fibroblasts were FN, COLI, COLIV, COLVI, versican, tenascin‐C, and decorin,^44^ while FN, COLI, COLIII, LN, decorin, and biglycan were the major constituents in the ECM from marrow stromal cells.^45^ Bae et al. found that fibroblast‐derived matrix, preosteoblast‐derived matrix, and chondrocyte‐derived matrix exhibited respective unique compositional and structural feature with distinct microenvironment for osteogenic differentiation.^46^

In the present study, we generated two kinds of biomimetic ECM from preosteoblasts and osteosarcoma cells to represent the physiological status and pathological status undergoing tumor, respectively (Figure 1). Denser fibers and higher Young's modulus were found in BM‐ECM than in OS‐ECM. During the experiments, we also found the BM‐ECM was thicker and easier to be reserved after decellularization compared to the OS‐ECM. Moreover, the GAG and collagen content were increased in the BM‐ECM. Osteoblasts were the main secretory cells of bone matrix. The balance between osteoblasts and osteoclasts maintained the dynamic stability of bone matrix assembly. During bone regeneration, osteoblasts were the key active cells to generate new collagens as well as bone matrix mineralization via osteoblastic differentiation. For osteosarcoma cells, their main activities were proliferation and invasion into bone matrix, which might lead to a decrease of the core matrisome secretion and an increase of the matrisome associated proteins. Besides the basic physical and chemical characterization of the dECMs, the ECM protein components are crucial for molecular mechanism of dECM matrix function.

Reviewing the previous literatures, the composition and mechanical properties of the ECM have been considered as drivers of tumor growth, local invasion, and dissemination of cancer cells to distant metastatic sites for breast cancer.^47^ More and more researchers focused on the essential role of ECM as a potential therapeutic strategy.^48^ Some key ECM proteins during breast cancer tumorigenesis have been discovered. Collagens, the most abundant component of the tumor ECM, were reported to regulate the breast cancer initiation and metastasis, including collagen I,^49^ collagen VI,^50^ and collagen XII.^51^ Chemotherapy was further demonstrated to induce metastasis via its effects on ECM composition, which might contribute to the recurrence of breast cancer.^10^, ^52^ Fatherree et al.^10^ reported chemotherapy‐induced collagen IV abundant in tumor ECM and promoted invasion via activation of Src and focal adhesion kinase, while Guarin et al.^52^ found chemotherapy‐induced collagen V abundant in liver ECM and increased cancer cell invasion via α1β1 integrin and MAPK signaling. Besides the direct composition investigations, Kay et al. reported metabolic reprogramming was also involved in halting the production of a pro‐tumorigenic ECM by targeting collagen synthesis.^53^ Moreover, the ECM in different metastatic organs (brain, lungs, liver, and bone marrow) revealed distinct metastatic niches as detected by proteomic profiling, and SERPINB1 was evaluated as a crucial ECM protein in brain metastasis.^11^ Nowadays, researchers mainly focus on the composition of ECM secreted by tumor cells and associated stromal cells, and the mechanism of ECM proteins on breast cancer cell invasion and metastasis. The biomimetic dECM system generated in the present study provided a convenient in vitro model to analyze the key ECM proteins for breast cancer bone metastasis. The deficiency of this study is that the molecular mechanism of the interactions between breast cancer cells and bone matrix has not been clarified, especially the key ECM proteins. Comparing the differential proteins in the two kinds of dECMs by protein mass spectrometry will help to discover the key ECM proteins and their functions in the process of bone metastasis of breast cancer.

To investigate the effect of bone mimetic dECMs on breast cancer cell migration, we designed several experiments ingeniously under different conditions (Figures 3 and 4). In most research, wound healing and transwell assay were used to analyze the cell migration under a specific factor basically the same. However, in the present study, both methods were used to clarify the different aspects of cell migration. On one hand, wound healing was used to evaluate the migration of cells on dECMs (Figure 3). For wound healing assay, the cells were cultured on dECM‐coated plate with the complete confluence. On the next day after cell adhesion, the cells and dECM were removed after scratching, as illustrated in Figure 3a. The migration of the cells cultured on dECMs was activated because of the dECM stimulation. The cells on BM‐ECM migrated quicker than on OS‐ECM, even to the uncoated area, which was caused by the activation of dECM niche. On the other hand, the transwell assay was used to evaluate the recruitment of the breast cancer cells to the biomimetic dECMs (Figure 4a–f). For the transwell assay, the cells were cultured on the uncoated surface on the top layer of the insert membrane, when the lower layer of the insert membrane was coated with dECMs. After that, the cells migrated through the holes in the insert membrane under the attraction of the dECM. Taking together, the BM‐ECM was demonstrated to promote the migration of breast cancer cells either on the dECM or to the dECM.

Increasing works showed dECM tumor models effectively recapitulated native ECM components and crucial communications between cancer cells and ECM, providing significant advantages over currently available in vitro testing platforms.^54^, ^55^ Cell‐derived dECMs offered highly economical platforms to detect the influence of ECM in tumor progression because of their highly controllable nature and have been recently used for the in vitro breast cancer model development.^48^, ^56^ For example, Lourenco et al. generated a conditioned dECM from adipose stromal cells under the stimulation of gastric cancer cell media, which exhibited a fibrotic arrangement with the increase of aligned collagen fibers and fibronectin deposition. The generated dECM was demonstrated to promote cell proliferation, clustering, and migration of cancer cells.^57^ Nayak et al. engineered a biomimetic PCL scaffold coated with dECM from cancer‐associated fibroblasts, which enhanced breast cancer cell attachment and viability.^58^ Compared to the dECM from single cell type, we developed a distinguished biomimetic dECM system with dECMs from two kinds of cells to monitor different bone matrix niches. The results showed the dECM system efficiently distinguished the low‐metastatic breast cancer cells and high‐metastatic breast cancer cells because the tendency of cell adhesion and migration was significantly different on the two kinds of biomimetic dECMs. The most interesting part was that the dECM system could detect early metastatic breast cancer cells in vivo with colonization on the BM‐ECM scaffold. Though the accurate mechanism was unclear right now, the biomimetic dECM system provided a smart platform with native microenvironment to discover the communications between cancer cell and matrix. Most importantly, the generated dECM system can provide a promising method for the early prediction of metastatic breast cancer cells.

Even though, there are some limitations to the present studies: (1) The dECMs were derived from two different species. The normal dECM was from mice, while the osteosarcoma dECM was from a human source. Despite this, 4T1 from mice and MDA‐MB‐231 from human showed the same trend of cell migration on dECMs in vitro, which indicated the generated dECM system could be applied to both mice and human. One possible reason might be the conservation of key proteins in human and mouse dECM. By now, we have no idea whether the human origin of dECM is better to distinguish the metastatic ability of human breast cancer cells. The effect of dECM species on cell activities needs more investigation in the future. (2) Although the dECM system exhibits excellent clinical translation potential for early detection of metastatic breast cancer cells, the breast cancer cells applied here were immortalized cell lines. It is better to study the cell activities of primary breast cancer cells from patients for clinical application next.

## 5 CONCLUSIONS

In summary, the distinguished biomimetic ECM system provided two different cell microenvironment, bone mimetic niche and bone tumor mimetic niche. Compared to tumor mimetic niche (OS‐ECM), the structure of native bone mimetic niche (BM‐ECM) was denser with more affinity to tissue plates. The ECM components in BM‐ECM were detected with more collagens and GAGs than in OS‐ECM. The most interesting was that the developed ECM system could efficiently distinguish the metastatic ability of breast cancer cells. Metastatic breast cancer cells tended to adhere and migrate on BM‐ECM, while low metastatic breast cancer cells tended to adhere and migrate on OS‐ECM. In the nude mice model with the implantation of biomimetic ECM‐coated scaffolds and tumor cells, BM‐ECM was demonstrated to successfully detect the early metastatic breast cancer cells before substantial organ metastasis, compared to OS‐ECM and COLI. Overall, this work highlighted the significance of specific cell microenvironment in breast cancer metastasis. The constructed biomimetic ECM system with different niches should be a promising in vitro tumor model to distinguish the metastatic ability of breast cancer cells and to capture the early metastatic breast cancer cells, holding great potential for clinical translation.

## AUTHOR CONTRIBUTIONS

**Bowen Weng:** Data curation (lead); formal analysis (equal); investigation (lead); methodology (equal); writing – original draft (equal). **Mei Li:** Conceptualization (equal); data curation (equal); formal analysis (equal); funding acquisition (equal); investigation (equal); methodology (equal); validation (equal); writing – review and editing (lead). **Weilai Zhu:** Investigation (supporting); methodology (equal); software (equal); validation (equal); writing – review and editing (supporting). **Jing Peng:** Investigation (supporting); methodology (supporting); validation (equal); writing – review and editing (supporting). **Xufeng Mao:** Investigation (supporting); methodology (supporting); writing – review and editing (supporting). **Yanan Zheng:** Investigation (supporting); writing – review and editing (supporting). **Chi Zhang:** Investigation (supporting); writing – review and editing (supporting). **Senhao Pan:** Investigation (supporting); writing – review and editing (supporting). **Haijiao Mao:** Methodology (equal); resources (equal); writing – review and editing (equal). **Jiyuan Zhao:** Conceptualization (lead); data curation (equal); formal analysis (lead); funding acquisition (lead); project administration (lead); supervision (lead); writing – review and editing (lead).

## CONFLICT OF INTEREST STATEMENT

The authors declare no conflicts of interest.

## Supporting information


**DATA S1.** Supporting Information.

## Data Availability

The original data supporting these findings are available at any time upon request to the corresponding author. The data are not publicly available due to privacy considerations.
