# Supplementary material for: Distinguished biomimetic dECM system facilitates early detection of metastatic breast cancer cells
Source: Bioeng Transl Med. 2023 Sep 1;9(1):e10597. doi: 10.1002/btm2.10597 (PMC10771560; doi:10.1002/btm2.10597)

**Fig. S1** Original data of western blots (WB) in Fig. 1.


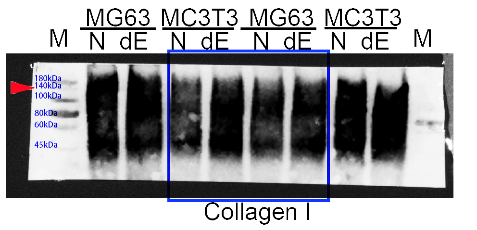

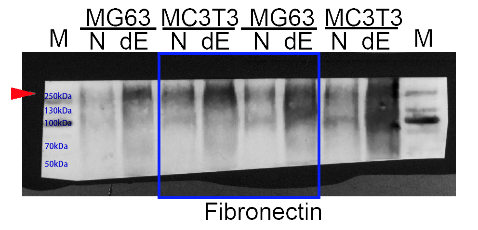

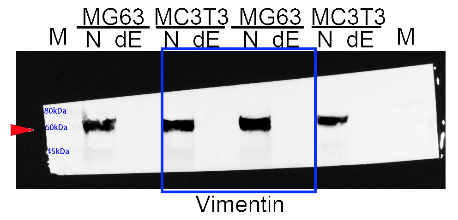

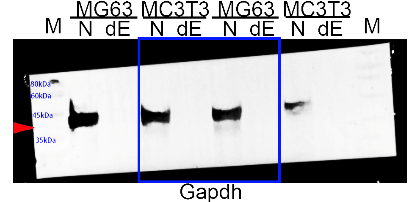

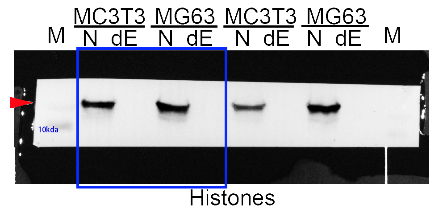


**Fig. S2** Original data of western blots (WB) to assess the protein levels of CXCR4 in 4T1 cells after culturing on different ECM on day 2. The membrane was incubated in CXCR4 primary antibody, following with appropriate secondary antibody incubation. The images were captured with gel documentation imaging system (Clinx Science Instruments Co., Ltd, Shanghai, China). The membrane was captured under white light model, while the WB bands were captured by ECL model at the same time and the same position. Subsequently, the membrane and the WB bands were merged. The membrane was washed with the eluent (Beyotime Biotech. Inc, Shanghai, China), and incubated with β-actin primary antibody, following with appropriate secondary antibody incubation and image capture. M: marker lane. After stripping, the marker was much weaker.


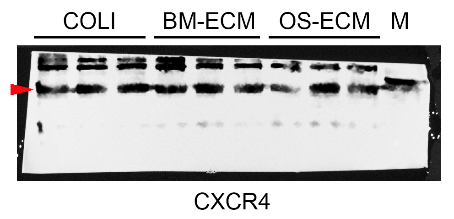

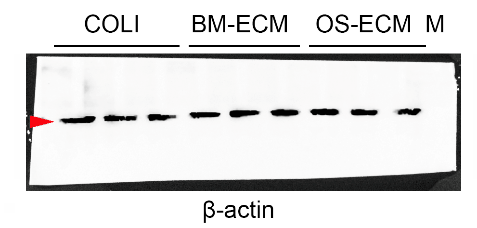
(4T1 cells, day 2)


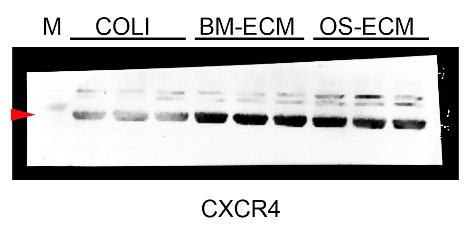

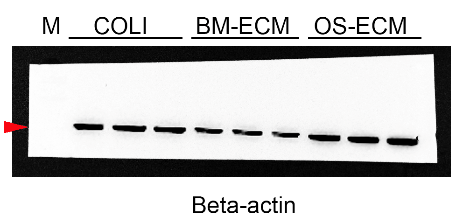
 (4T1 cells, day 4)


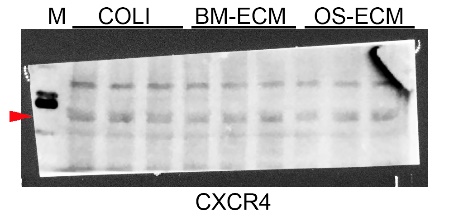

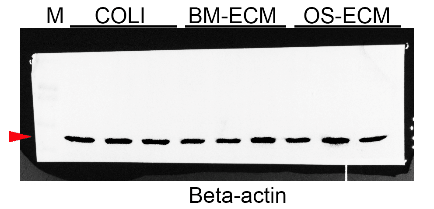
(MDA-MB-231, day 2)


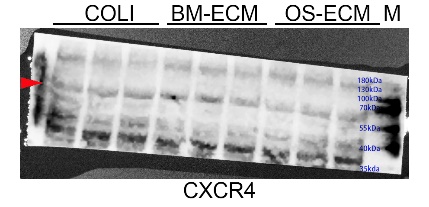

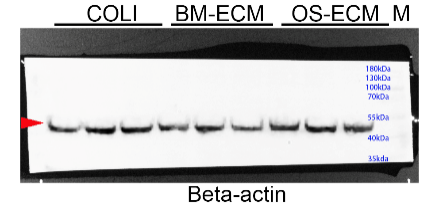
 (MDA-MB-231, day 4)


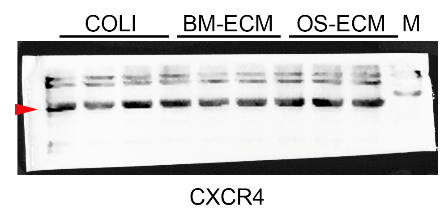

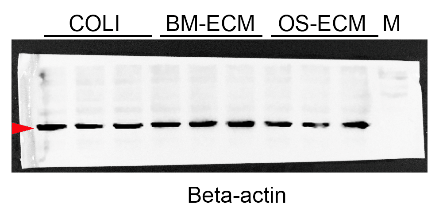
 (MCF7, day 2)


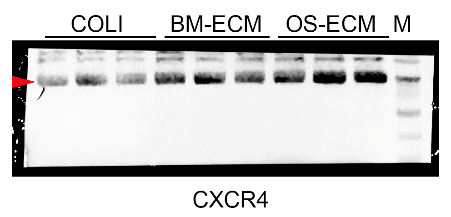

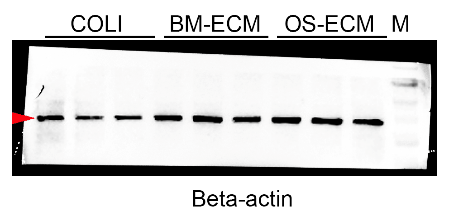
 (MCF7, day 4)


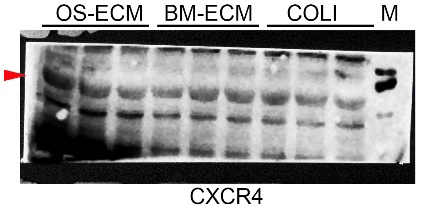

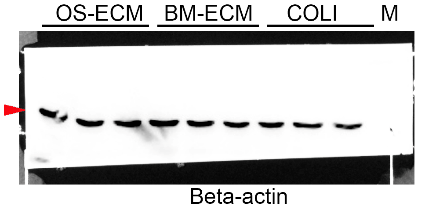
 (HCC1937, day 2)


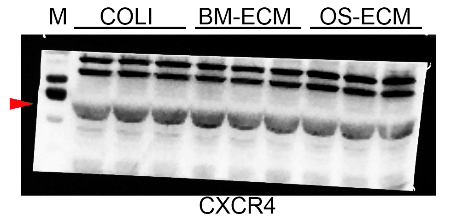

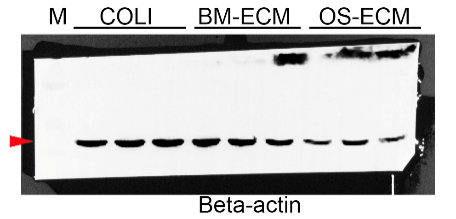
 (HCC1937, day 4)

**Fig. S3** Original data of western blots (WB) to assess the protein levels of EMT markers (E-cadherin, Vimentin and Beta-actin). For 4T1 cells, the membrane was first incubated in E-cadherin primary antibody, following with appropriate secondary antibody incubation. The images were captured with gel documentation imaging system (Clinx Science Instruments Co., Ltd, Shanghai, China). The membrane was captured under white light model, while the WB bands were captured by ECL model at the same time and the same position. Subsequently, the membrane and the WB bands were merged together. The membrane was washed with the eluent (Shanghai Epizyme Biomedical Technology Co., Ltd, Shanghai, China), and incubated with Vimentin primary antibody, following with appropriate secondary antibody incubation and image capture. Then, the same membrane was stripped again, and incubated with Beta-actin primary antibody, following with appropriate secondary antibody incubation and image capture. M: marker lane.


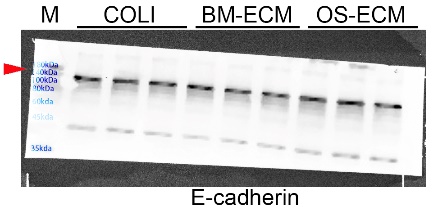
 (4T1, day 1)

(Exposed at first with substrate)


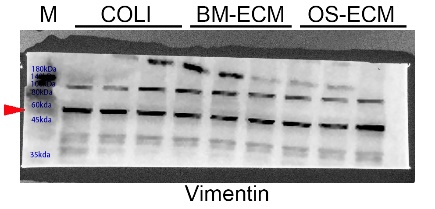
 (4T1, day 1)

(The membrane was washed with the eluent (Shanghai Epizyme Biomedical Technology Co., Ltd, Shanghai, China), and incubated with Vimentin primary antibody. Residual bands were observed)


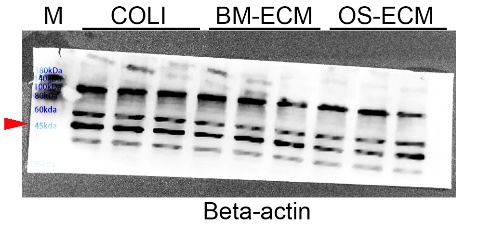
 (4T1, day 1)

(The membrane was washed with the eluent (Shanghai Epizyme Biomedical Technology Co., Ltd, Shanghai, China), and incubated with Beta-actin primary antibody. Residual bands were observed)


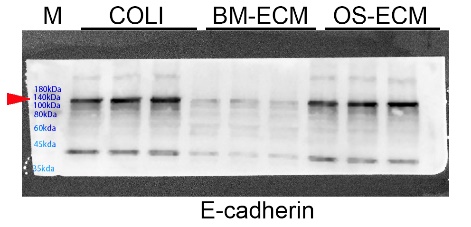
 (4T1, day 2)

(Exposed at first with substrate)


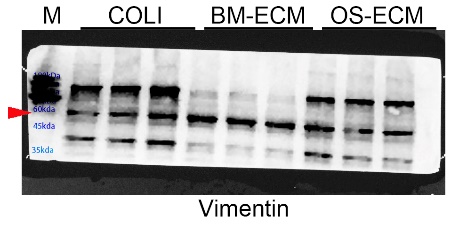
 (4T1, day 2)

(The membrane was washed with the eluent (Shanghai Epizyme Biomedical Technology Co., Ltd, Shanghai, China), and incubated with Vimentin primary antibody. Residual bands were observed)


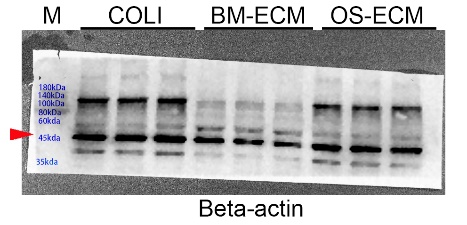
 (4T1, day 2)

(The membrane was washed with the eluent (Shanghai Epizyme Biomedical Technology Co., Ltd, Shanghai, China), and incubated with Beta-actin primary antibody. Residual bands were observed)


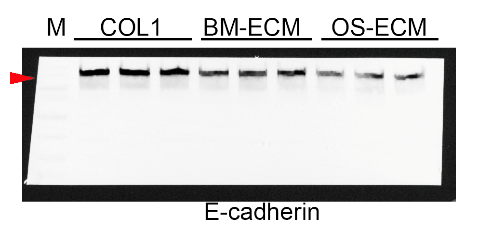
 (MCF7, day 2)

(Exposed at first with substrate)


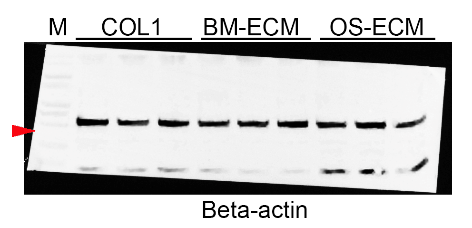
 (MCF7, day 2)

(The membrane was stripped and incubated with Beta-actin primary antibody. The eluent solution was from Beyotime Biotech. Inc, which was more efficient with less residual bands)


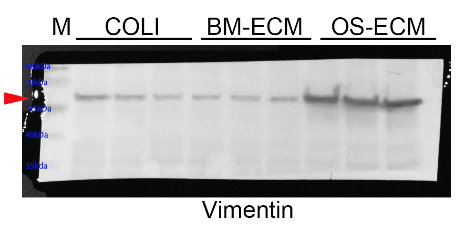
 (MCF7, day 2)

(Because the molecular weight of Vimentin and Beta-actin was very close, Vimentin was exposed in another membrane with the same loading protein content.)

**Fig. S4** Young’s modulus of empty plates, COLI coated plates and dECM coated plates (BM-ECM and OS-ECM). For collagen coating, the empty plate was immersed in the COLI solution (10 mg/mL) overnight. After ventilation and drying, the COLI coated plates were stored at 4 degrees for storage. The COLI coating was very thin and the Young’s modulus of COLI coated plates was similar to the empty plates. The Young’s modulus of empty plates and COLI coated plates was much higher than dECM coated plates (BM-ECM and OS-ECM).


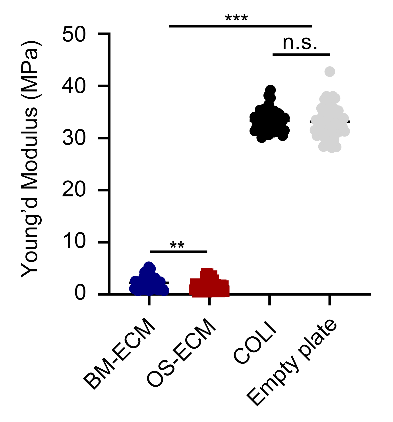


**Fig. S5** Highly metastatic breast cancer cells (4T1) and lowly metastatic breast cancer cells (MCF7) were seeded on COLI, BM-ECM and OS-ECM respectively. The mRNA levels of *TFF1* were measured by quantitative real-time PCR. Triplicate experiments were performed for each group. *, *P*<0.05; **, *P*<0.01; ***, *P*<0.001.


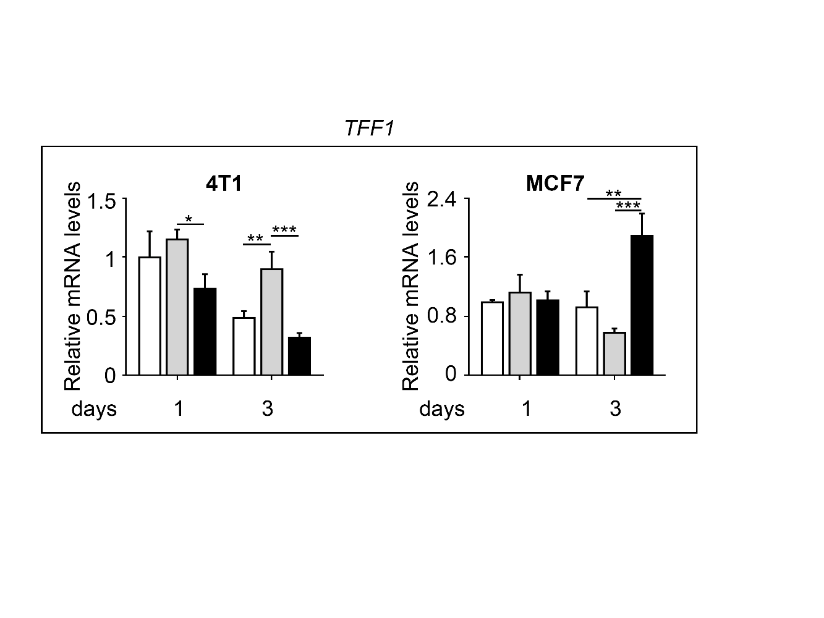


**Fig. S6** MCF7 cells were cultured on COLI, BM-ECM or OS-ECM respectively as in Fig. 7B, following with the Live/Dead cell staining. According to the instructions of the Live/Dead TM cell imaging kit (Cat. R37601, Invitrogen, Carlsbad, CA), the cells were incubated in equal volume of 2X working solution and imaged under a fluorescence microscope. The live cells were stained as green, and the dead cells were stained as red. The cells treated with 20 μg/mL puromycin were used as a control to show the dead cells.


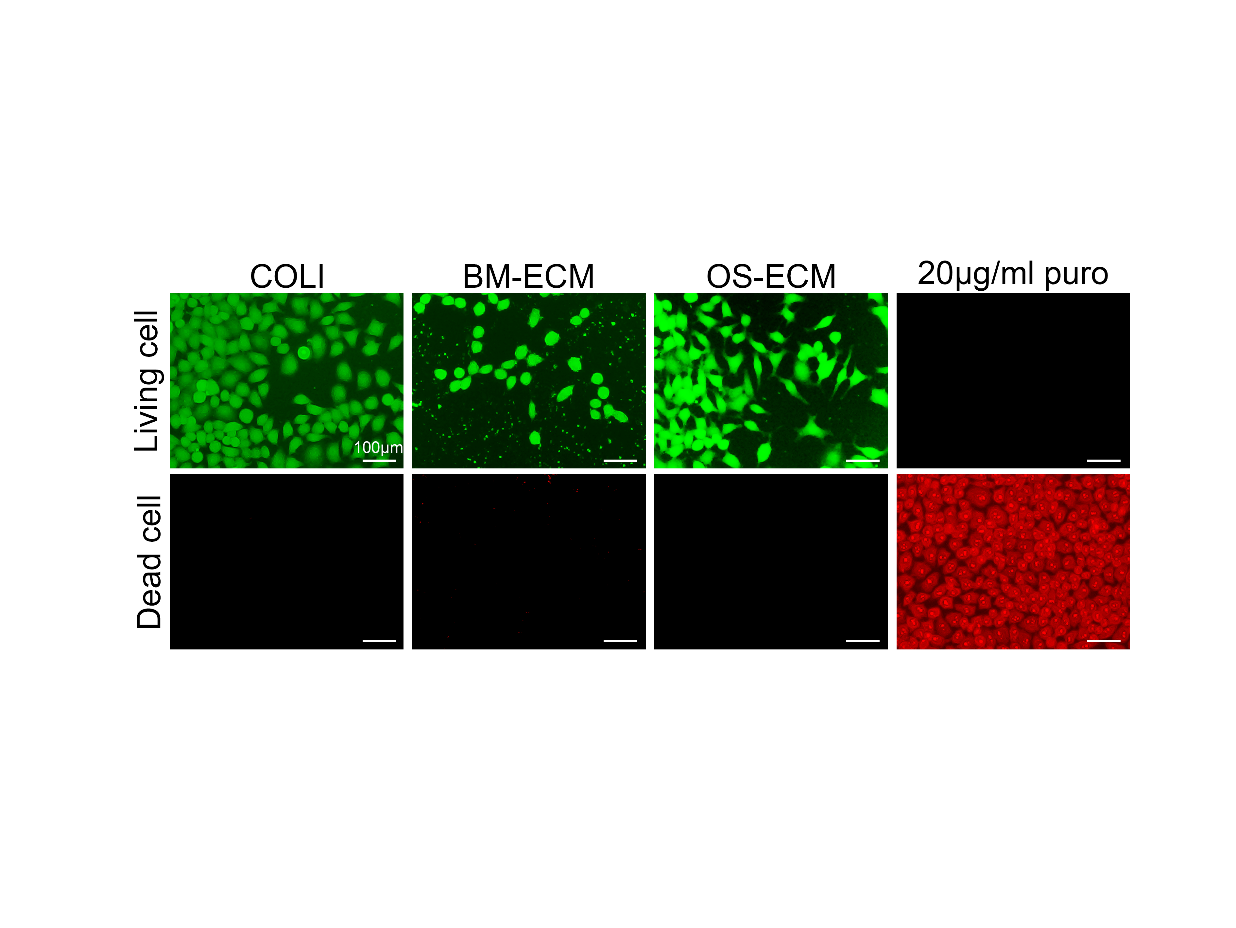


**Fig. S7** The mRNA expression of the essential transcription factors as measured by real-time PCR. The breast cancer cells with high metastatic ability (4T1 (A-C)) and low metastatic ability (MCF7 (D-F)) on different dECMs were assayed at different time points (12, 24 and 48 h). Snai1 (A&D), Twist (B&E) and Slug (C&F) expression were assessed.


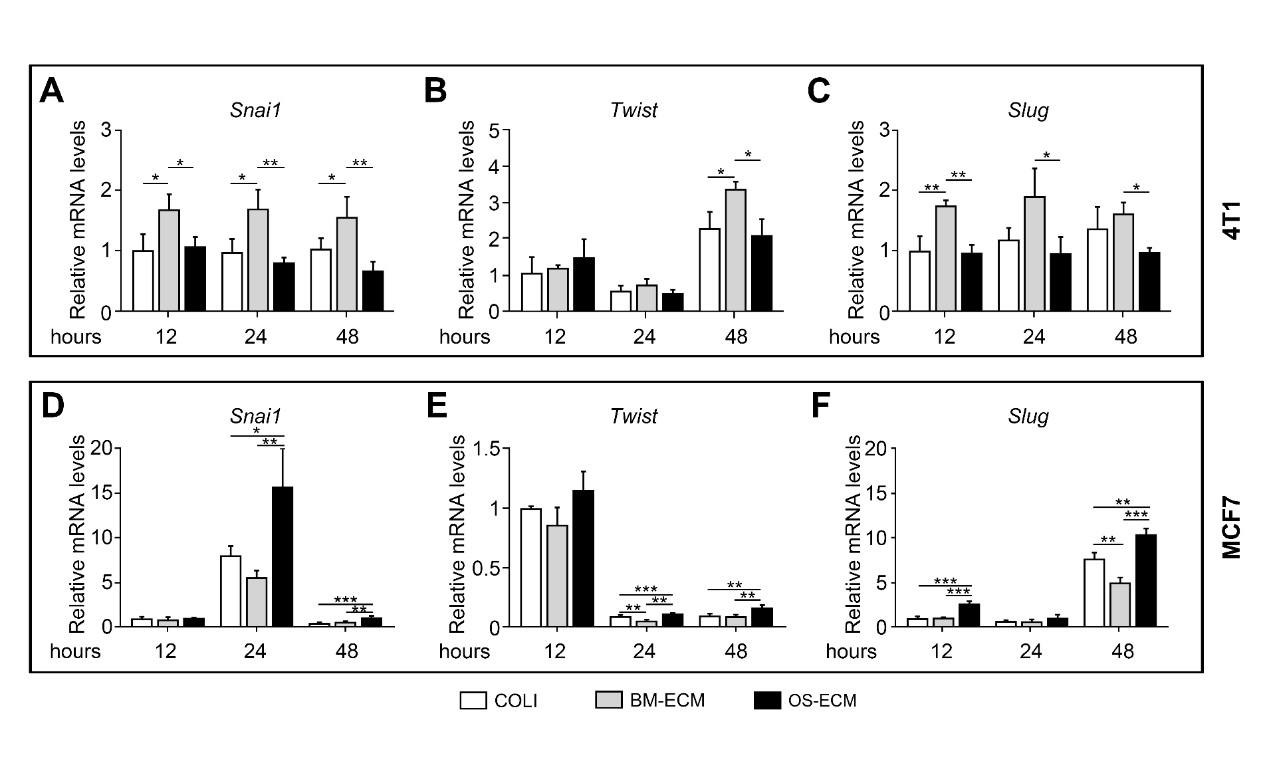


**Fig. S8 The luminescence scan of potential metastatic organs.** After 10 days of RFP-4T1 cells injection, the BM-ECM scaffolds and potential metastatic organs (heart, liver, spleen, lung, kidney, and bone) were captured at the same time under the same threshold. Significant signals were observed in BM-ECM scaffolds, while no positive signal was observed in the potential metastatic organs, which indicated early detection of metastatic breast cancer cells by the distinguished biomimetic dECM system before distant organ metastasis.


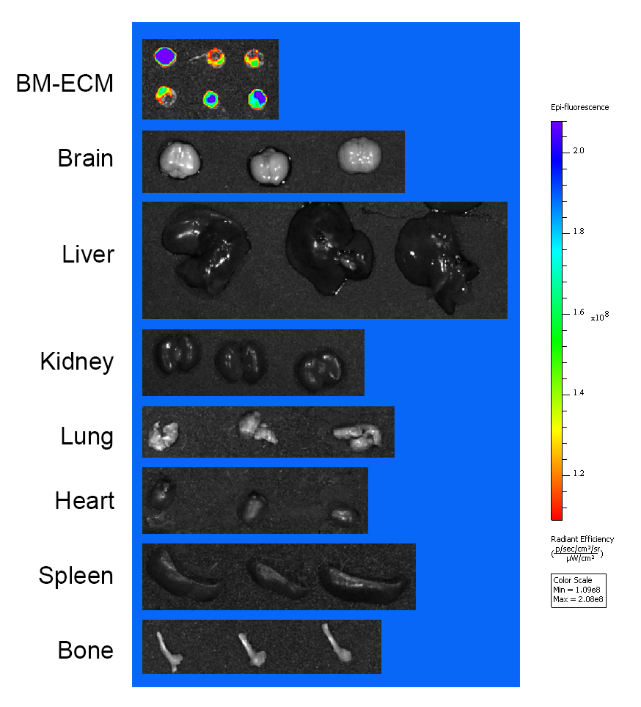

Supplement: Supplementary file 1 — DATA S1. Supporting Information. [file BTM2-9-e10597-s001.docx]
